# Mg-Alloys for Forging Applications—A Review

**DOI:** 10.3390/ma13040985

**Published:** 2020-02-22

**Authors:** Nikolaus P. Papenberg, Stefan Gneiger, Irmgard Weißensteiner, Peter J. Uggowitzer, Stefan Pogatscher

**Affiliations:** 1 LKR Light Metals Technologies Ranshofen, Austrian Institute of Technology, A-5282 Ranshofen, Austria; stefan.gneiger@ait.ac.at; 2 Christian Doppler Laboratory for Advanced Aluminum Alloys, Chair of Nonferrous Metallurgy, Montanuniversität Leoben, A-8700 Leoben, Austria; irmgard.weissensteiner@unileoben.ac.at; 3 Chair of Nonferrous Metallurgy, Montanuniversität Leoben, A-8700 Leoben, Austria; peter.uggowitzer@mat.ethz.ch (P.J.U.); stefan.pogatscher@unileoben.ac.at (S.P.); 4 Department of Materials, Laboratory of Metal Physics and Technology, ETH Zürich, 8093 Zürich, Switzerland

**Keywords:** magnesium alloys, forging, literature review, overview

## Abstract

Interest in magnesium alloys and their applications has risen in recent years. This trend is mainly evident in casting applications, but wrought alloys are also increasingly coming into focus. Among the most common forming processes, forging is a promising candidate for the industrial production of magnesium wrought products. This review is intended to give a general introduction into the forging of magnesium alloys and to help in the practical realization of forged products. The basics of magnesium forging practice are described and possible problems as well as material properties are discussed. Several alloy systems containing aluminum, zinc or rare earth elements as well as biodegradable alloys are evaluated. Overall, the focus of the review is on the process control and processing parameters, from stock material to finished parts. A discussion of the mechanical properties is included. These data have been comprehensively reviewed and are listed for a variety of magnesium forging alloys.

## 1. Introduction

Magnesium, the world’s lightest structural metal, has attracted much attention in recent years. Possible applications of this material are closely connected to its low weight and good specific mechanical properties. Therefore, many studies focus on the positive influence which Mg alloys can have on the lightweight construction of products in the transport sector such as automobile and aircraft components. Intensive efforts to reduce weight made in the past are well known and have repeatedly resulted in substantial changes in the choice of materials (e.g., from steel parts to multi material mixes) and designs (e.g., introduction of space frames). Naturally, these different potential applications require materials with a multitude of properties. These have to be investigated, understood and tested before they can finally be reliably produced on an industrial scale.

Forged parts in general are typically used for structural applications with high demands on reliability, functionality and mechanical properties. These qualities are particularly important in transportation, which makes these industries a main customer of forged parts. The use of Mg provides the additional benefit of reduced mass and enables new ways of light weight design. Therefore, forged Mg parts seem to be especially well suited for applications in the transport sector.

Magnesium alloys, their properties, applications and possible developments have been described in various studies throughout the years [1,2,3,4,5]. The corrosion behavior, which is an integral part of many applications, has been reviewed extensively by Esmaily et al. [6] and the related topic of coatings for Mg products has been described by Gray and Luan [7]. The precipitation behavior of Mg alloys as the main source of strength in many materials is reviewed by Nie [8]. An overview of forming by extrusion processes and the resulting properties is given by Zeng et al. [9]. Although there are works on Mg alloys for forging applications [10,11,12,13,14], this aspect is far from being as profoundly reviewed as the above topics are.

A short introduction about the forging of Mg in Europe is given in the works of Sillekens et al. [10,11,12], which discuss the benefits and challenges of industrial implementation in detail. Ovsyannikov [13] briefly describes the industrial forging practice and the products made of various Mg alloys. In a publication of Dziubińska et al. [14], the application of Mg forgings in transport applications is reviewed and examples for produced components are given. General information on the forging of light metal alloys in general can be found in the works of Shan et al. [15]. Hartley and Pillinger describe the simulation of forging processes [16] and Hawryluk and Jakubik [17] present a work on forging defects.

**Figure 1:**
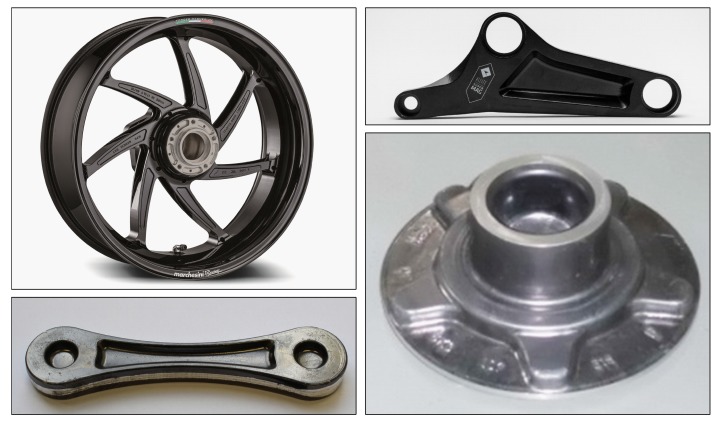
Various Mg forged parts, showing (u.l.) a motorbike rim produced by Brembo S.p.A. under Marchesini trademark, (u.r.) a bike suspension link made by ALLITE${}^{®}$ Inc., (b.l.) laboratory scale piston rod forged by Light Metals Technologies Ranshofen and (b.r.) a wheel hub produced by Institut für Metallformung, University Freiberg. Reproduced with the kind permission of: Brembo S.p.A., ALLITE${}^{®}$ Inc., Light Metals Technologies Ranshofen and TU Bergakademie Freiberg.

While forged Mg products are presented in various scientific studies, industrial applications are still mostly confined to high-priced applications for sports and military use. Nevertheless, these products highlight the possible benefits and performance of forged Mg alloys. Unfortunately, however, the leap towards the use of Mg forgings in the mass market of consumer goods has not yet been successful. Examples of some forged parts made from Mg alloys are shown in Figure 1, which depicts forgings either commercially available or used in research and development.

In the course of the development of Mg alloys many different properties, such as flow behavior, mechanical properties and texture have been investigated and the understanding of these materials has improved steadily. Here we give an overview of the scientific literature on Mg forging. The alloys and the processing parameters used are presented and the product range is described. By presenting the efforts made in the field, the variety of available materials and the current state of the art are illustrated.

In order to provide a comprehensive overview of the most relevant aspects of Mg forging applications, we start with a brief history of the use and development (Section 2) as well as an introduction about the basics of Mg forging (Section 3). Studies using various Mg alloys for forging are discussed in Section 4, Section 5, Section 6, Section 7, Section 8 and Section 9. Subsequently, a short conclusion is drawn in Section 10. Tables containing processing parameters and mechanical properties of various alloys can be found in the Appendices Appendix B and Appendix C, respectively.

The main online sources used for the literature research concerning this review were: Scopus, Google Scholar, Web of Science, Espacenet and Google Patents. This work does neither include publications about powder or thixo forging nor forgings for the sole purpose of grain refinement (e.g., multi axial forging).

## 2. History

Magnesium was already known and has been scientifically investigated throughout the 19th century. However, industrial production only slowly emerged at the beginning of the 20th century, starting out in Europe, where castings and parts were already displayed 1909 in Germany. From there it crossed into the US where production picked up in the 1920s and accelerated in the 1930s [18]. The process for the production of primary Mg varied, depending on the usable local resources, ranging from electrolytic (Downs process) to carbo-thermic (Hansgirg process) and silico-thermic (Pidgeon process) [19].

The benefits of this new light weight structural material stayed not unnoticed to other assurgent industries. To cover the rising demand, the suppliers, differing by size and country, started to increase their output by producing primary Mg, Mg alloys and parts. Manufacturers from this time, still well known today, are Dow Chemical (US), Magnesium Electron (GB) and I. G. Farbenindustrie (Germany). Main customers of Mg products were aircraft and automobile industries, where engine and structural parts as well as wheels and rotors were used. As reported by Gann [18] in 1929, Mg accounted for 50% of light metals used in German aircraft work. The Berlin Transportation Co. used Mg wheels in their motor coaches since 1926, not only to improve driving behavior but also to increase tire life.

Nevertheless, one should not forget that much of this development was pushed by the military on the eve of World War II. This is well visible in the breakdown of Mg production after 1945. Regardless, the industry searched for civil applications and production was able to grow again from the 1950s onward [2,20]. The main Mg producers were the US until the end of the 1990s, after which China started up their own production. Today, China is by far the biggest producer of primary Mg worldwide, producing more than 80% of the available material.

The main quantity of Mg is currently used as alloying element for Al alloys, for desulphurization in steel production and as Mg casting alloys, while wrought Mg alloys and products account only for a small fraction of the Mg in use. Nevertheless, the forming of Mg has always been a topic of interest for the scientific community and industrial applications which demand for good specific mechanical properties.

Already in 1924, the forging of Mg parts for aviation was discussed briefly by Portevin and deFleury [21]. There, the importance of heated dies is mentioned and the authors conclude that Mg alloys can be forged easily, maybe even better than high strength Al alloys. In the overview on Magnesium presented by Gann [18] in 1930/31, the importance of moderate forming-speeds is highlighted and press-forging is recommended in lieu of drop-forging. While artificial ageing was known for Mg cast products, it seems not to be applied to forgings. The degree of deformation, on the other hand, is highlighted as important for the mechanical properties.

In 1939 Haughton [22] describes the use of an isothermal forging process. Moreover, the increased formability of upset forging stock and the improved mechanical properties of forgings produced with forging steps of subsequently lowered temperatures were mentioned.

While, throughout the years, the main alloying systems for Mg are the Mg-Al, Mg-Al-Zn and Mg-Mn systems, more complex systems (e.g., containing Ce, Ag, Pb and Be) have been investigated as well [19]. In 1950, Grube et al. [23] reported on their investigations of Mg-Ce forging alloys, which were analyzed regarding their high temperature properties and creep behavior. The review on Mg-Li alloys done by Frost [24] in 1962 features forgings as well, besides the description of various forming processes and the comparison of the resulting mechanical properties.

Of special interest for the production of forgings might be the report by Shaw et al. [25] concerning the effect of lubrication on the forming behavior, and the work of Sabroff et al. [26]. The investigation on lubricants used for the forming of various materials from 1955 showed that the best results for Mg alloys were achieved by graphite-based lubricants [25]. The report of Sabroff et al. [26] from 1964, a manual on forging of all kinds of metals, describes the forging practice of Mg alloys as well. It covers the description of the most important alloys and gives information on forming behavior, lubricants, grain size control and trimming.

## 3. Basic Aspects of Magnesium Forging

In general, forgings have better mechanical properties than cast parts and show favorable microstructural flow in loading direction if produced appropriately. This originates from a reduction of casting defects, closing of pores, refinement and breaking of primary phases as well as grain refinement and material flow while forming. Forgings are thought to show the best overall mechanical properties of all Mg products [27].

The use of Mg alloys in light-weighting shows its benefits particularly in bending applications where substantial increases in stiffness, strength and reduction of instabilities are possible with equal part weight. When heavier metals are exchanged for Mg alloys, it can be beneficial to modify the geometry, but this is not always necessary. Often, the part has already been designed in a way that the originally used material can be substituted directly with Mg alloys without a critical degradation in mechanical properties [27].

### 3.1. Alloy Designations

To describe the chemical composition of an alloying system or an alloy, designation systems are widely used. While various such systems exist, the one preferentially used in scientific literature is the ASTM Standard Alloy Designation System (B951-11) and also this work uses this system.

The ASTM Standard Alloy Designation System consists of four parts, the principal alloying elements, which are defined by one letter each, are the first part. In the second part the rounded-off percentages (wt%) of the respective elements are given. The third and fourth parts are the number of standardization (starting with the letter A and omitting O and I) and the temper designation. Regrettably, the ASTM does not provide designations for all available alloying elements, therefore the designations used by the authors cited are adopted in this work. An overview of the most common alloying elements and the respective designations based on the ASTM system are given in Table 1 and a selection of temper designations is presented in Table A2 in the Appendix B.

### 3.2. Forming Behavior

The forming behavior and suitability of Mg alloys for forging processes can be investigated with a multitude of tests, the most common are tensile and compression (upset) testing and also backwards extrusion.

Compression, upsettability or upsetting tests can be conducted with a multitude of testing parameters (e.g., temperature, strain rate, etc.) and sample shapes. The resulting deformation of the sample is controlled by lubrication, die design, sample shape and material behavior [28]. Most commonly, testing is done on a cylindrical billet between two flat dies. The billet is compressed till either cracks appear or to a predefined strain. Thereby, the forming behavior, possible surface defects and necessary deformation force can be measured directly. The microstructure and (depending on the sample size and analysis method) the mechanical properties of the deformed samples can be analyzed as well.

**Figure 2:**
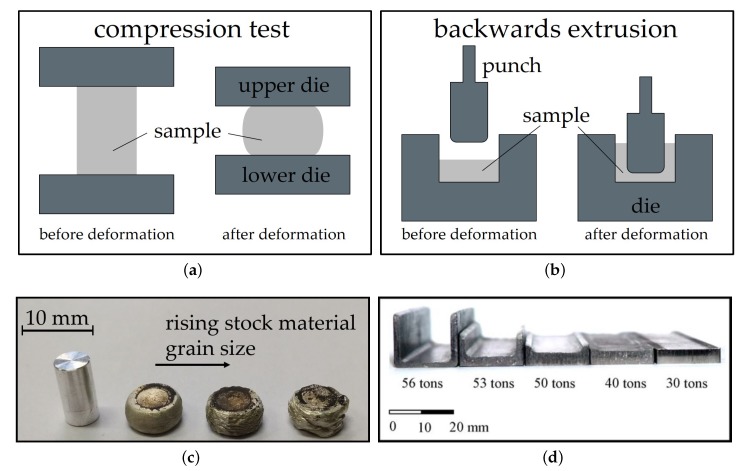
Schemes of simplified testing set-ups for (**a**) compression testing and (**b**) backwards extrusion; (**c**) AXM lean alloy compression samples with varying stock material grain sizes tested at 400 °C. Picture (**d**) shows parts manufactured from AZ31 by backwards extrusion at 250 °C with varying forming load [29], reproduced with permission from Elsevier.

Backwards extrusion is a relatively simple testing set-up that can be implemented both experimentally and by simulation. The material is pressed into a die by a punch and the layout leaves space for the compressed material to flow into the opposite direction of the punch. Thereby a cup or a comparable form is shaped. The height of the walls of the part is dependent on the material flow behavior, lubrication and used forming load. In terms of complexity, backwards extrusion can be considered an intermediate step between compression testing and more complex die forgings. Compared to compression testing, backwards extrusion testing accomplishes higher degrees of deformation, higher hydrostatic stresses and exhibits a more complex material flow. In backwards extrusion The testing schemes and sample shapes for compression testing and backwards extrusion are depicted in Figure 2.

**Figure 3:**
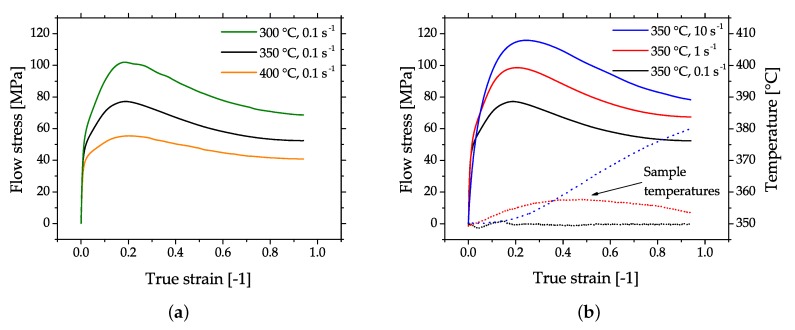
Flow curves of cast and homogenized AZ31, obtained by cylinder compression testing. The plots show the flow stress (full lines) of samples tested with (**a**) varying temperatures and (**b**) varying strain rates. The dotted lines in (b) show the increasing sample temperatures with increasing strain rate. No temperature compensation was done for the shown flow stress values.

The flow behavior of Mg alloys in compression testing is characterized by softening after reaching the peak stress. Microstructurally, this behavior is due to as dynamic recrystallization (DRX) and it is the most beneficial deformation mechanism for a successful forming of Mg parts. In case of higher forming speeds the flow stress usually increases, but this can be mitigated by an increase of the material temperature which causes a decrease in flow stress. A typical example of this behavior is given in Figure 3, where flow curves of cast and homogenized (425 °C for 24 h) AZ31, measured by cylindrical compression tests are shown.

When tensile testing is applied to layout a process, it is important to take into account that the necessary forging pressure might exceed the tensile strength by far [26]. Although Mg parts can be formed by hammer forging, die forging with hydraulic presses is commonly used. The main reasons for this are the reduced ductility, increased flow stress and cracking sensitivity at higher forming speeds (occurring in hammer forging). Mg alloys, like many other materials, show improved forming behavior in case of increased hydrostatic stresses, because free surfaces are especially prone to cracking while forming. Therefore, a closed-die process facilitates optimal forging conditions.

### 3.3. Forging Stock

The stock material should be well homogenized to disperse eutectic phases and it should exhibit a small grain size, as grain size is a main aspect of the forming behavior in Mg alloys besides temperature and forming speed [30]. Cracking of Mg parts with coarse grains can be easily seen in the case of higher forming speeds, for example, in the behavior of the flash. Therefore, it is not surprising that often pre-deformed stock (mainly extruded) is used for forging. It is well known that extruded Mg has a high degree of anisotropy, which strongly influences the flow behavior during forging as well as the mechanical properties in the finished part. This can be taken into account by providing increased deformation into the transverse direction, thereby improving the usually low transversal ductility [26].

### 3.4. Die Design

The die design used for Mg alloys is comparable to that applied for Al alloys. If the same dies are used, the differing processing parameters and thermal expansion coefficients might result in slightly different part sizes at room temperature (RT). Depending on the alloy it might be necessary to use additional forming steps for Mg. To achieve a good surface quality of the forged parts, the dies should have a smooth surface, which also eases metal flow while forming [30]. Magnesium can only be forged at elevated temperatures; the dies should therefore be made of materials with sufficient high-temperature strength. According to Behrens et al. [31], 1.2344 (X40CrMoV5-1), 1.2365 (32CrMoV12-28), 1.2367 (X38CrMoV5-3), 1.2714 (56NiCrMoV7) as well as other conventional low-alloy hot-work tool steels are commonly used [30]. For layout purposes a number of recommended radii for corner and fillet of Mg forgings are listed in Reference [27].

### 3.5. Temperature Control

Temperature control of the billet and the dies is essential in the forging process. While forging usually takes place well below the melting temperature and, therefore, fire hazard is greatly reduced, care has to be taken to avoid excessive overheating and hot spots while heating the material. The billets should be heated uniformly to achieve good forging results and avoid failures like shear or hot cracking [30]. The temperature of the forging stock depends on the material used (alloy, as-cast, homogenized or extruded) but also on the die temperatures, forming speed, billet shape and size, number of applied forging steps and degree of deformation. These factors all play a role when looking at the forming window of a product; other criteria might be mechanical or microstructural properties. The die temperatures can either promote underfilling or surface cracking if too hot or cold, respectively [26]. Controlling the forging temperature is also a way to influence the grain size of the produced part. To keep the grain size small, the forming temperature can be reduced in each forging step. Magnesium alloys are known for static recrystallization after deformation; to prevent this, the finished parts can be quenched in water. An overview of frequently used stock and die temperatures according to Reference [30] is given in Table A3 in the Appendix B.

### 3.6. Lubrication

Lubrication is an important part of every forming practice. For Mg forgings graphite-oil or graphite-water suspensions are usually used, depending on the die temperature. For higher temperatures oil-graphite suspensions are suitable. In all cases the carrier fluid evaporates from the heated dies and a thin graphite film remains on the surface [30].

According to the study on lubricants on AZ80A, conducted by Shaw et al. [25], very good results have been achieved with both, a mix of graphite also with powdered MoS${}_{2}$ in water. For a further improvement of penetration into die cavities not only the dies can be lubricated but the stock material as well. This is realized by vapor blasting or etching (using acetic acid) of the billet and a subsequent dipping into the lubricant before heating it to forming temperature. According to Sabroff et al. [26] care should be taken to keep the flash regions—where friction is desired—free of lubricant.

### 3.7. Trimming

Trimming of forged Mg parts can be either done at the minimum forging temperatures or the flash can be removed by sawing at room temperature (RT). Warm trimming might pose some problems with bending or warping of the part, therefore this is only done if the flash regions are sturdy enough. Flash removal by band saw at RT is common if only small quantities of parts are produced. Mg-alloys often show brittle fracture behavior in case of trimming at room temperature using a trimming press [26,30]. In some cases, parts might then be ruined as the brittle fracture of the flash extends into the part itself.

### 3.8. Machining

According to Reference [27], Mg alloys can be machined easily with or without lubricants (coolants) at high speeds. Compared to other structural metals like Al, the tool wear and power required for machining is reduced and the parts obtain a smooth finished surface. Lubricants (mineral oils) are mainly used as coolants to decrease possible part distortion and chips ignition. Increased risk of fire can be the case if cutting speeds over 5 m$s^{-1}$ are applied and feeds are smaller than 0.02 mm. Fine cuts produced by finishing might also be ignited by sparks if handled improperly.

### 3.9. Microstructure and Mechanical Properties

**Figure 4:**
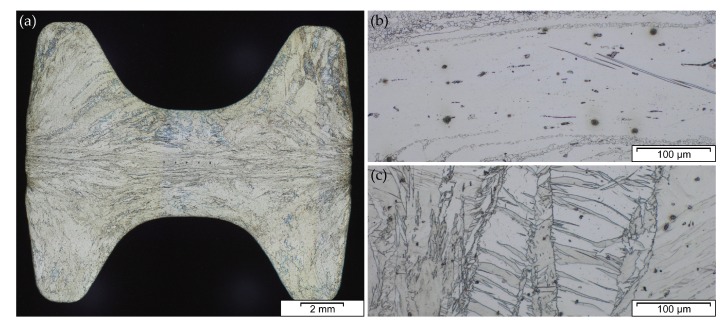
Microstructure of an AZ31 variation (AZ31 containing 0.3 Ca and 0.2 Y) in as-forged condition, formed at 425 °C stock temperature and 280 °C die temperature at a ram speed of 10 mm$s^{-1}$, showing (**a**) cross-section of forged part, (**b**) sample center, (**c**) sample rim.

The microstructure and subsequently the mechanical properties of Mg forgings can vary excessively within a part. The final microstructure depends on temperature, degree of deformation and forming speed. It might be composed of twinned grains, fine recrystallized grains, necklace structures, shear bands and combinations thereof in the same part. This behavior is pronounced in as-forged parts. The example given in Figure 4 stems from a laboratory-scaled piston rod [32]. The varying degrees of deformation are well visible in the microstructure of the cross-section. In the sample center a combination of deformed and fine recrystallized grains, a so-called necklace structure, is present. On the sample rim, having a lower degree of deformation, large, heavily twinned grains are prevalent. In the case of a subsequent heat treatment or slow cooling of the parts, recrystallization progress depends on available energy and nucleation points, for example, twin and grain boundaries.

**Figure 5:**
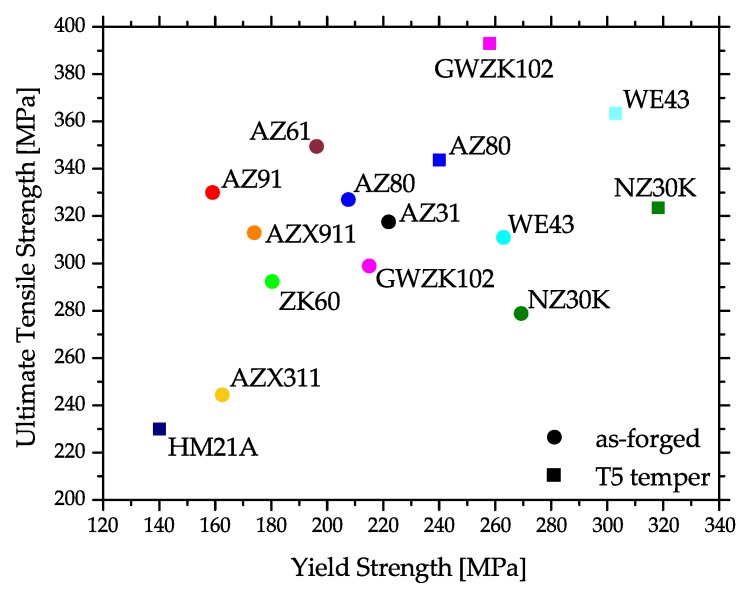
Tensile properties of various Mg alloys, showing as-forged and artificially aged (T5) values at room temperature (RT). The graphic shows mean values calculated from various scientific sources. A more detailed overview is given in Appendix C.

Corresponding to this behavior, the mechanical properties may differ considerably throughout the part. This is the case for strength properties, but especially for ductility, where the difference between twinned and recrystallized microstructure may be considerable. An overview of literature values of yield strength (YS) and ultimate tensile strength (UTS) values of various Mg alloys at RT is given in Figure 5. A more detailed summary is given in Appendix C, where tensile properties and process information is compiled.

### 3.10. Heat Treatments

Heat treatments for Mg alloys are similar to those known for other materials (i.e., Al). The well known steps of homogenization, solution heat treatment and artificial ageing, or a combination thereof, can be used for various alloys in the production of wrought Mg parts.

Some confusion might exist when looking at the parameters applied for heat treatments of Mg casting products, where the solution heat treatment can last for hours. This originates in the purpose of the heat treatment, which primarily aims to dissolve primary phases after casting. Adequate parameters (time and temperature) should also be used for wrought products in the homogenization heat treatment before forming. The solution heat treatment of wrought alloys, on the other hand, has a much shorter duration. This is the case because the alloying elements should already be well dispersed in the material and excessive grain growth of the usually fine grained and/or deformed microstructure should be avoided. While quenching is not necessarily done after homogenization it might very well be necessary after a solution heat treatment to prevent premature precipitation of hardening phases. Artificial ageing times and temperatures have to be adapted to the alloy used. Especially the ageing duration can vary excessively. For example, rare earth containing alloys may have ageing times of multiple days [33].

An overview of possible heat treatment temperatures and times for various alloys is given in Table A4.

## 4. Magnesium Alloys for Forging Applications: Methodology

In the following sections various Mg alloys used for forging applications will be discussed, concentrating on the scientific works published in the last 10 years. The literature is evaluated with regard to the alloy type under investigation and divided into sections corresponding to the alloy systems: Mg-Al (Section 5), Mg-Zn (Section 6), rare earth containing alloys (Section 7), biodegradable alloys (Section 8) and various other alloying systems (Section 9). These chapters each contain a short introduction for the different alloys described, as well as phase diagrams, CALPHAD calculations and microstructural pictures, where meaningful. Subsequently scientific works investigating the respective alloys are discussed individually. Because of the diverse use of alloying elements, the investigated works are sorted in relation to the topic addressed in the corresponding literature, that is, papers about the effects of Ca in an AZ91 alloy will be discussed in the section about aluminum calcium alloys (AX-System).

This structure was chosen because even small changes of used forging stock, forming parameters and die design can result in considerable differences in forming trial results, mechanical properties and microstructural features. Readers can therefore more easily select the most interesting or beneficial study for their own work.

Additionally, a compilation of applied processing and heat treatment temperatures (Appendix B) as well as mechanical properties and the respective processing parameters of the discussed scientific works (Appendix C) are given in the appendices of this review.

## 5. Forging of Magnesium Alloys Containing Aluminum

**Figure 6:**
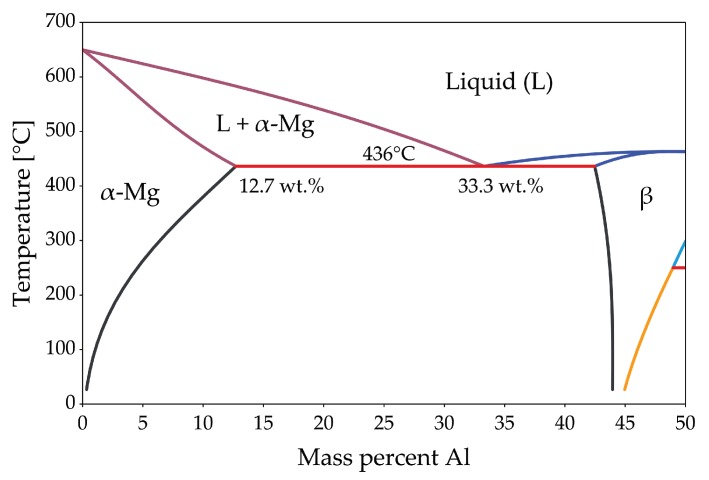
Phase diagram of the Mg-Al binary system. For further information, see Appendix A.

Aluminum was one of the first and is still the most important alloying element for Mg. More than 90% of all Mg structural applications are made from alloys within the Mg–Al system (mainly AZ91 and AM60) [34]. Al increases strength, hardness and castability of Mg alloys and allows precipitation hardening of alloys containing more than 6 wt.% [35]. Mg-Al shows eutectic behavior with a relatively high solubility limit of Al in Mg with 12.7 wt.% at the eutectic temperature and 0.5 wt.% at room temperature. The eutectic reaction L →α (Mg) + β (Mg${}_{17}$Al${}_{12}$) takes place at 437 °C. As derived from the phase diagram shown in Figure 6, the β−phase can be completely dissolved and exploited for precipitation hardening if the Al-content is lower than 12.7 wt.%. Nevertheless, at high Al-contents and with high amounts of eutectic due to segregations upon casting, long heat treatment times can be necessary for complete dissolution of Al into the Mg-matrix.

In the following the different sub-systems of the Mg-Al base alloys are reviewed.

### 5.1. Mg-Al-Zn-System

For technical applications, Mg–Al alloys are often alloyed with low amounts of Zn (<1 wt.%), which further increases their strength at room temperature and their corrosion resistance [35]. Mg–Al–Zn is the most important alloying system for casting and forming applications with Al-contents ranging from 3 to 9 wt.% and Zn-contents lower than 1 wt.%. Additionally, Mn is used in minor contents likewise for increasing the corrosion resistance [35]. Typical alloys used for forging are AZ31 (Mg–3%Al–1%Zn), AZ61 (Mg–6%Al–1%Zn), AZ80 (Mg–8%Al, <1%Zn) and AZ91 (Mg–9%Al–1%Zn) where the latter is commonly used as a casting alloy but nevertheless forgeable.

The diffusion speed of Al in Mg is rather low leading to a divorced eutectic at the grain boundaries consisting of β–phase embedded in a supersaturated α–matrix. With increasing Al content, the amount of brittle β–phase at the grain boundaries increases, resulting in low ductility and limited formability. Therefore, a low Al content is usually preferred for forming operations. Nevertheless, if high strength is required, higher amounts of Al are common.

Nominal chemical compositions of commercial Mg-Al-Zn alloys are given in Table 2.

**Figure 7:**
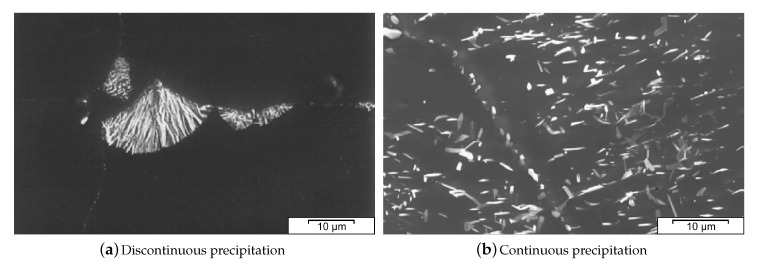
Scanning electron micrography (SEM) micrographs of homogenized and artificially aged AZ91, showing (**a**) discontinuous and (**b**) continuous precipitation [39]. Reproduced with permission from Elsevier.

An example for an AZ forging alloy is AZ80, which has been investigated by Sager et al. [38]. It was shown that the forming behavior of this alloy can be improved by a homogenization of the forging stock. Thereby the brittle β−Mg${}_{17}$Al${}_{12}$ phase is reduced and a supersaturated α−Mg solid solution is created. Precipitation of Mg${}_{17}$Al${}_{12}$ can occur after casting or can be used as a means of age hardening (T5 and T6 states) and it takes place continuously or discontinuously. In the discontinuous case the precipitating lamellae grow from Al rich areas (e.g., eutectic regions) into the α−Mg grains. It is usually assumed that continuous precipitation is preferable in terms of precipitation size, homogeneity and the thereby resulting in improved mechanical properties. Accordingly, the precipitation behavior of AZ91 has been investigated in detail by Braszczyńska-Malik [39]. Both types of precipitation are shown in Figure 7.

The increasing material strength with rising Al content has also been confirmed in a comparative study of AZ31, AZ61 and AZ91 done by Madaj et al. [40]. Two different geometries, a piston rod and a plate, were forged, heat treated and analyzed by hardness measurements. The forging stock was homogenized at 380 °C to 420 °C for 15 h. The forming itself took place at 300 °C to 350 °C, depending on the alloy, with a die temperature of 150 °C to 170 °C. A recrystallization heat treatment (420 °C for 3 h) of the formed parts was tested on all alloys. The different alloys performed satisfactorily during forging and all parts were formed without defects. Finally, an improved hardness with increasing Al-content was observed in all sample conditions, both for the initial and for the as-forged and the heat treated material. The highest values were reached by the as-forged AZ91 parts.

#### 5.1.1. AZ31

AZ31 is well established as wrought alloy in the scientific community and has become something of a benchmark material in a multitude of applications. Therefore, many publications deal with the forming and forging of this alloy. A broad range of stock materials, from cast to highly deformed stock, as well as various processing parameters have been investigated throughout the years.

**Figure 8:**
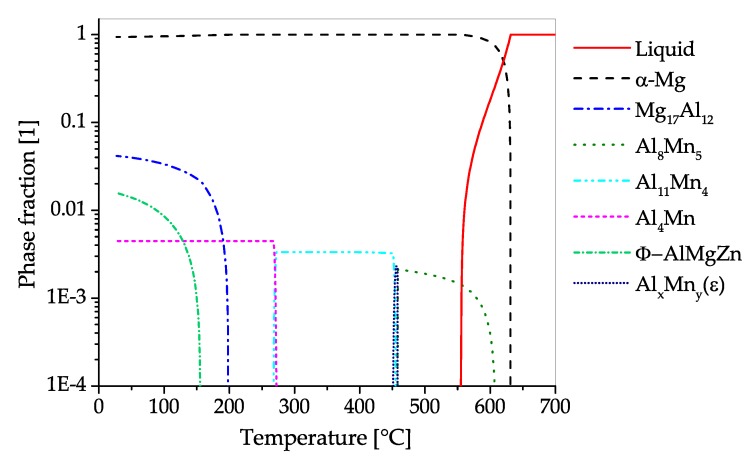
CALPHAD calculations (for further information see Appendix A) of AZ31 in a temperature range of 25 °C to 700 °C, showing phase fractions of $10^{-4}$ to 1.

The CALPHAD calculation of AZ31 (Figure 8) shows the fraction of present phases in the equilibrium state over temperatures, ranging from fully liquid to room temperature. The predominant precipitating phase is Mg${}_{17}$Al${}_{12}$, which can nevertheless be dispersed into the solid solution over a broad range of temperatures. Additionally, a minor amount of Al-Mn-phases with changing stoichiometry can start to precipitate during solidification. Zinc is present in AZ31 mainly in solid solution. Nevertheless, a ternary AlMgZn phase can be formed at low temperatures.

**Figure 9:**
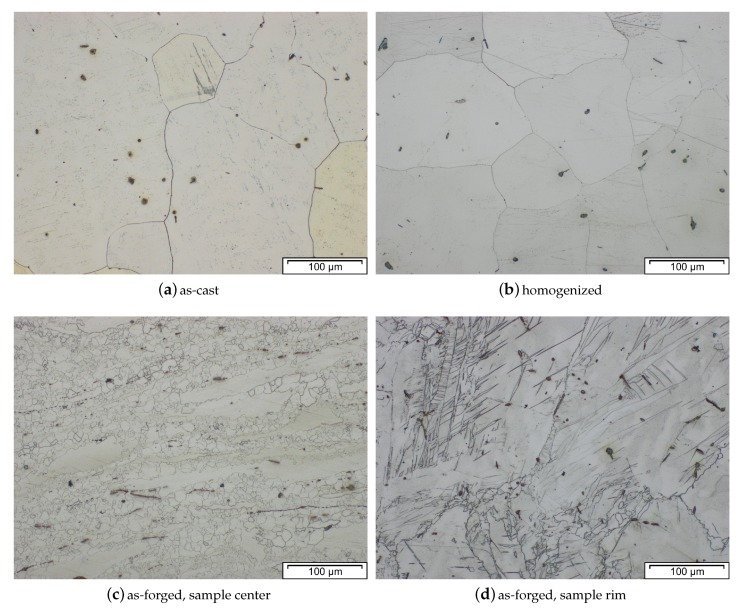
Microstructure of AZ31 in consecutive processing steps: (**a**) as-cast, (**b**) homogenized at 415 °C for 24 h. Forging took place using a material temperature of approx. 425 °C, die temperatures of 280 °C and a ram speed of 10 mm$s^{-1}$. The as-forged microstructures of parts quenched in water are shown in: (**c**) center area having high degree of deformation and (**d**) flange area with a lower degree of deformation.

The microstructure of an AZ31 alloy for various processing steps is shown in Figure 9. The material was cast into a steel mold and shows no distinctive features after casting. A homogenization heat treatment (415 °C for 24 h) was done to dissolve possible phases (Mg${}_{17}$Al${}_{12}$) at the grain boundaries and to ensure an even distribution of the alloying elements before forming. For the forging, the same sample geometry as depicted in Figure 4 was applied. The as-forged microstructure is mostly devoid of intermetallic phases and there are differences visible depending on the degree of deformation. The sample center (Figure 9c) consists of a banded structure with fine recrystallized grains and elongated remains of the original grains. In contrast, the microstructure on the sample rim (Figure 9d) shows a high fraction of large, intensely twinned grains, showing that neither the temperature nor the degree of deformation was sufficient to start recrystallization of these grains.

The forgeability of AZ31 cast material was analyzed by Skubisz et al. [41]. There, the die forging trials (stock temperatures 200 °C to 300 °C, tool temperature 200 °C and ram speed 1 mm$s^{-1}$) were conducted with two different stock geometries, varying the height to diameter ratio (h/d). The best results were found in the h/d = 0.8 samples formed at 300 °C. The samples produced at h/d = 2.5 and other temperatures cracked while forming.

It is shown in multiple publications that extruded AZ31 forging stock has a superior formability at lower temperatures compared to cast AZ31. Chino et al. [42] for example conducted upsetting tests in a temperature range from 50 °C to 400 °C at an initial strain rate of 0.004 $s^{-1}$. The authors concluded that a forging temperature of at least 300 °C is advisable for samples with good surface quality. An increase of YS of the samples forged at lower temperatures was attributed to the accordingly decreasing grain size.

In the work of Wong [43], testing took place between 300 °C to 500 °C at 0.001 to 1 $s^{-1}$, which yielded comparable results. Also, an increased surface roughness and a certain sensitivity to low temperatures and higher feasible forming speeds of the cast pre-material have been observed. An improvement of workability was noticed after a homogenization heat treatment (450 °C for 5 h) of the cast stock. The compression behavior of the extruded material was investigated as well, showing an anisotropy along the extrusion direction.

Such anisotropic forming behavior of extruded AZ31 was also recorded by Dai et al. [44]. In their work, samples from 3 material directions were compressed at 200, 300 and 400°C with a strain rate ranging from 0.01 $s^{-1}$ to 10 $s^{-1}$. They concluded that, dependent on the loading direction, the deformation processes favor either slipping or twinning.

In a comparison of extruded and continuously cast AZ31 and AZ80 presented by Viehweger et al. [45], no differences in the forging behavior were observed. While the flow curves (compression tests at 300, 350, 400 and 450°C, with 0.1, 1 and 10 $s^{-1}$) and final microstructures varied in the diverse samples and alloys, the forging of simple geometries, using a punch speed of 1 mm$s^{-1}$ to 40 mm$s^{-1}$ at various temperatures, was consistently successful. Skubisz et al. [46] investigated the material behavior of AZ31 at high forming speeds using a screw press. Compression testing and a FEM simulation were used for process layout and the forging took place at temperatures ranging from 170 °C to 350 °C. The extruded forging stock showed better formability when compared with cast material. Moreover, an improved formability was found in case of increased hydrostatic pressure while forming.

Graf et al. [47,48] used compression testing (250 °C to 450 °C at 1 $s^{-1}$ to 10 $s^{-1}$) and FEM simulation to support the die forging process of a wheel hub. To partially eliminate the brittle β to phase the cast material was homogenized at 430 °C for 6 h prior to forming. Testing of both, the cast and the extruded stock, took place at 250, 350 and 450°C at ram speeds of 1;10mm$s^{-1}$ and with a die temperature of 200 °C. The analysis of the forged parts showed distinct microstructural differences dependent on the forming parameters, that is, forming speed, temperature and degree of deformation. In Reference [48] the same authors compared the forgeability of extruded AZ31 with AZ61 and AZ80. It was found that AZ31 had the worst forming behavior of the three alloys in the tested range of 250 °C to 450 °C at a ram speed of 10 mm$s^{-1}$. AZ60 could be formed well when using temperatures over 350 °C, showing a fine microstructure, attributed to the recrystallization, stimulated by Mg${}_{17}$Al${}_{12}$ particles. The best results were archived in AZ80, which was found to be well formable even at 250 °C.

Confirmatory results are shown in the work of Behrens and Schmidt [49], where AZ31 (as-cast as well as extruded) and extruded AZ61 were compared by forging pulley wheels. The results of either AZ31 stock was unsatisfactory in the presented forging process (300 °C to 400 °C at 12 mm$s^{-1}$ to 160 mm$s^{-1}$ and at a die temperature of 350 °C). The extruded AZ61 stock was deformed at the same temperatures, however, the produced parts showed a better form filling behavior and higher surface quality. Subsequent tensile testing of parts forged from AZ31 and AZ61 showed an improved UTS but a reduced YS, which were claimed to be dependent on forming temperature and speed.

Cui et al. [50,51] applied compression tests and FEM simulations to configure the forging process and die geometries of two different spur gear designs. In Reference [50] presumably extruded AZ31 was forged in two steps (at 0.5 mm$s^{-1}$) to a bevel gear. In the first step, the material was heated up to 300 °C while the die temperature was 275 °C, in the second step the temperature was lowered to 290 °C and 265 °C for stock and die respectively, to improve the mechanical properties of the finished parts. The part formed in Reference [51] was a straight spur gear, using extruded AZ31 as forging stock. In the supporting simulation, the resulting grain size of the formed part was calculated with help of the Zener-Hollomon parameter (ZH-parameter). Based on these results, the forging took place at 300 °C and 0.1 mm$s^{-1}$.

Extruded blanks of AZ31 and ZK60 were used by Poerschke [52] to forge disks with a rim and spoke design via a two-step forging process. The forgings were done at 315 °C to 375 °C using a screw and a hydraulic press, subsequently the microstructural behavior, showing typical banded structures, and mechanical properties of the produced parts were investigated. The parts made from ZK60 showed better tensile properties throughout the process chain when compared to the parts made of AZ31 material.

The recrystallization behavior of AZ31 and AZ61 alloy plates was analyzed by Watanabe et al. [53] to understand the grain size evolution in the forming process. The samples were deformed by upset forging in a range of 200 °C to 400 °C with an initial strain rate of 0.033 $s^{-1}$. The results were used to correlate the ZH-Parameter to the grain size of the dynamically recrystallized microstructure. It was found that the initial grain size of the material has an influence on the grain size of the recrystallized grains, and that the ZH-Parameter increases in case of decreasing grain sizes after recrystallization.

AZ31 plates, as also extruded parts, usually exhibit a certain anisotropy in mechanical behavior and microstructure. This is discussed in the work of Rao et al. [54] were a rib-web-shape was forged and analyzed. A processing map and a model using the ZH-parameter was established, based on results from isothermal compression tests (300 °C to 550 °C, 0.0003 $s^{-1}$ to 10 $s^{-1}$) and compared to the forming behavior of forged parts. Isothermal forging took place at temperatures ranging from 300 °C to 500 °C and 0.001 mm$s^{-1}$ to 10 mm$s^{-1}$. Anisotropic behavior was found to be reduced in the DRX regime of the process, as the authors concluded from the final sample shapes.

In the studies of Dziubińska et al. [55,56,57,58] AZ31 plates were used to produce various live sized brackets with ribs. For this purpose, the process was simulated and a newly designed, three slide forging press was used. The forging was done at a stock temperature of 410 °C and die temperatures ranged from 200 °C to 250 °C at 6 mm$s^{-1}$. After forming microstructures and mechanical properties varied, depending on the local strains applied in the forging process [56,58]. This was especially apparent in the tensile properties of the brackets, an example being the YS which varied between 220 MPa and 298 MPa in the same part. The concept, challenges and possible problems of the forming process using a three slide forging press are described in References [55,57].

**Figure 10:**
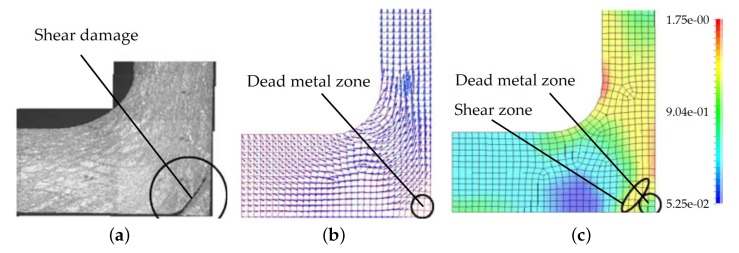
Comparison of shear damage in an AZ31 (**a**) forged part, formed at 250 °C, (**b**) finite element method (FEM) simulations showing nodal vector displacement and (**c**) plastic strain distribution [29]. Reproduced with permission from Elsevier.

Lim and Yong [29] used AZ31 plate material for backwards extrusion trials. Forming took place at 200, 250 and 300 °C with 0.25 $s^{-1}$ in a hydraulic press. The forging process was simulated and afterwards the microstructure and the material flows within the parts were investigated. It was found that shearing damage may occur in hardly deformed areas, so called dead metal zones (Figure 10).

Backwards extrusion of AZ31 with varying forming speed has been investigated by Matsumoto and Osakada [59] on cylindrical specimen. The AZ31 stock was annealed (350 °C for 1 h) before isothermal forming in the temperature range of 20 °C to 400 °C at an initial strain rate of 12 $s^{-1}$ at maximum. It was found that a decreased forming speed at the beginning of the deformation is beneficial to the forging. This is presumably caused by a more homogeneous temperature distribution within the sample.

In some work, forging trials were conducted on heavily pre-deformed stock material. Takara et al. [60] used AZ31 sheets (thickness 0.8 mm) to form a part with ribs perpendicular to the surface. Parts with varying lubrication conditions were formed isothermally at 350 °C at a speed of 0.005 mm$s^{-1}$. In a subsequent analysis the material flow in the rib area was investigated and it could be shown that the lubrication has a profound impact on the material flow when forming parts with such small cross-sections.

Lee et al. [61] used stock material, pre-deformed by equal channel angular pressing (ECAP) with a grain size of approximately 3 μm to forge an impeller without burr. Forming took place at 300 °C, applying a strain rate of 0.001 $s^{-1}$, which allowed to retain the small grain size of the forging stock. The as-forged microstructure was investigated and the micro hardness measured. While forming, the grain size increased, reaching 4 μm to 6 μm. The resulting hardness fluctuated at 60 HV and decreased with increasing grain size.

AZ31 was used by Kápustová and Bílik [62,63] to form a lever by closed die forging without flash. This forming process exhibits high hydrostatic stress and reduced material loss because of the missing forging flash, on the other hand it is primarily useful for the production of simple, rotary-shaped parts and requires a specifically shaped forging stock. The process and die layout was construed by FEM simulations, assuming die and material temperatures of 250 and 400 °C, respectively. The FEM calculations could be successfully verified by forming trials, where parts without defects were produced in this one step forging process.

Mróz et al. [64] used AZ31, plated with Al, to forge a bimetallic door handle. The stock material was produced by explosive welding, where an AA1050A tube was bonded to an AZ31 rod. Forming was done in three steps (bending and two forging steps) at 400 °C material temperature and die temperatures of 300 °C. A screw press capable of a tool speed of 30000 mm min was applied in this process. While there was no damage of the bimetallic bonding zone, the covering Al layer was unduly pressed into the forging flash. This was attributed to the low flow stress of the AA1050A cladding material at the high forming temperatures. As primary purpose of the Al coating is to protect the AZ31 center material from corrosion, the measured corrosion properties varied strongly, depending on the quality of the still existent Al surface layer. For mechanical testing AZ31 forgings without Al cladding were used. The finished forging showed improved properties (a YS of 234 MPa and 280 MPa UTS) as well as reduced grain sizes, when compared to the stock material.

The tension and compression behavior as well as fatigue properties of forged AZ31 were investigated by Toscano et al. [65,66]. As-cast stock material was homogenized at 450 °C for 3 h and formed directly afterwards. The material was open-die forged isothermally at 450 °C at a ram speed of 6.5 mm$s^{-1}$, producing so called ‘flatbread’ or ‘pancake’ samples in a single step. The tensile properties of the forged and cast material are compared and while a distinct increase in strength was found in the forged samples, the elongation was reduced. The forging also showed anisotropic mechanical behavior, which was verified by texture analysis. Strain controlled fatigue testing (R=−1) was done for both materials and it could be shown that the low cycle fatigue (LCF) life of the forged samples was increased. In accompanying microstructural analyses extensive twinning could be shown. Analyses of the fracture surfaces revealed the initiation of fatigue cracking to occur at intrusions-extrusions, surface pores, extension twinning and oxide layers. In a subsequent work [67], the same material was used to form a more complex part by closed-die forging at 275 °C and 20 mm$s^{-1}$. The strength of the formed part was found to be increased as well as the fatigue properties (load controlled, at R=−1) when compared to the cast forging stock.

Gryguc et al. analyzed the compressive [68] as well as the tensile and fatigue behavior [69] of extruded and forged AZ31. The extruded stock was compressed isothermally up to 85% engineering strain at 400 °C by applying strain rates of 0.002;0.02;0.075 $s^{-1}$. From the resulting pancake-shaped parts, as well as from the extruded stock, samples for compression, tensile and fatigue testing were machined. All tested mechanical properties were found to be improved by the forging process. This was attributed to a change of texture and the change from a bimodal grain distribution to a refined and equiaxed structure. The results were supported by investigations of the fracture surfaces of the fatigue samples, which showed a dimpled fracture surface for the forged samples in comparison to a terrace-like structure in the extruded ones.

#### 5.1.2. AZ61

The aluminum content of the alloy AZ61 lies in-between the two main representatives of forming and casting alloys, AZ31 and AZ91 respectively. It is considered as both, a casting as well as a wrought alloy. According to Reference [35] its Al content of 6 yields an optimum combination of ductility and strength. While this alloy can, in principle, be used in heat treated condition, this is usually not the case for forgings, which are mainly used in as-forged state [30].

**Figure 11:**
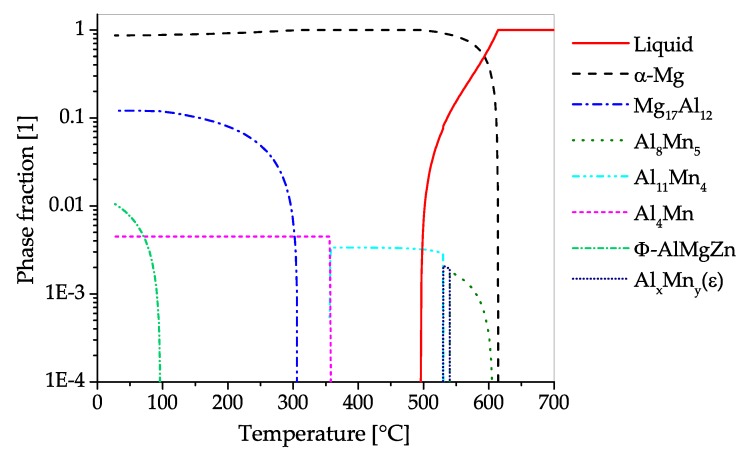
CALPHAD calculations (for further information see Appendix A) of AZ61 in a temperature range of 25 °C to 700 °C, showing phase fractions of $10^{-4}$ to 1.

The CALPHAD calculation (Figure 11), shows the fraction of present phases in AZ61 over the temperature range from fully liquid to room temperature in equilibrium state. As in AZ31, the predominantly precipitating phase is Mg${}_{17}$Al${}_{12}$, which can nevertheless be dissolved into the solid solution over a broad range of temperatures. Additionally, a minor amount of AlMn-phases with varying stoichiometry can be found in the alloy, which already begin to precipitate during solidification. Zinc is found mainly in solid solution, however, at low temperatures an AlMgZn-ternary phase can be formed.

Skubisz et al. [41] analyzed the forming behavior of wrought AZ61 (hot deformed to 0.83 effective strain) by die forging tests. Two different stock geometries (ratio of height to diameter: 0.8 to 2.5) were used for the deformation at stock temperatures of 150, 200 and 350 °C (tool temperature 200 °C, ram speed of 1 mm$s^{-1}$). Only the forging at 150 °C showed cracking. Nevertheless, all samples showed adequate surface quality.

Yoon et al. [70] investigated the forming behavior and process parameters of extruded AZ61 forging stock with compression testing and simulation. Forging trials were done at 350 °C with a speed of 32 mm$s^{-1}$. The stock material was pre-deformed by upsetting at room temperature (RT), up to an axial strain of 0.06 and 0.6 in order to compare the warm forming behavior of twinned versus recrystallized material. The produced parts were analyzed with regard to microstructure and mechanical properties. It was found that recrystallized forging stock increases the formability but reduces the achievable tensile strength in comparison to the twinned AZ61.

Various authors compared the forging behavior of AZ61 and AZ31 [48,49,53]. These studies are briefly described above, in Section 5.1.1 for AZ31. Madaj et al. [40] describes the forming behavior of AZ61 at a material temperature of 320 °C in a work comparing the formability of AZ31, AZ61 and AZ91, which is discussed in Section 5.1. A comparison of forging behavior and mechanical properties of AZ61, AZ80 and various TAZ alloys by Yoon and Park [71] is reviewed in Section 9.

#### 5.1.3. AZ71

Material made from the alloy AZ71 was advocated as forging stock for large parts that is, wheels by Fugita et al. [72]. They discussed the large grain size of Mg-Al alloy castings and the resulting problems for Mg forming products. The AZ71 alloy is reported to form a reduced grain size in casting, especially with additions of Sr and/or calcium cyanamide (CaNCN), making it a good candidate for forging without prior extrusion. Upsetting tests were conducted to investigate the forgeability in the range of 250 °C to 400 °C with 0.01 $s^{-1}$ to 10 $s^{-1}$.

**Figure 12:**
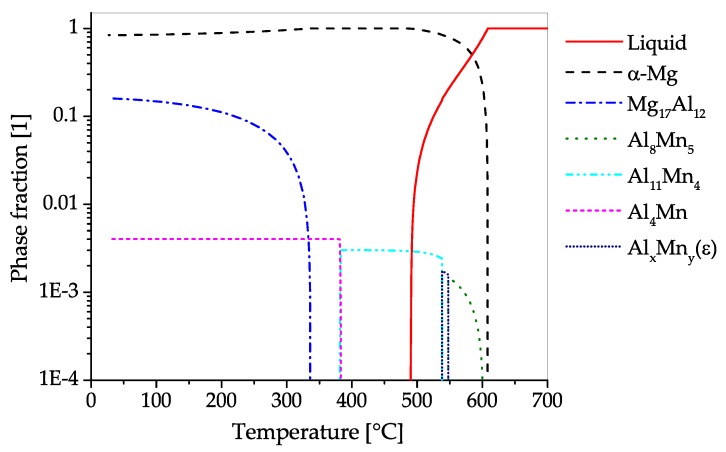
CALPHAD calculations (for further information see Appendix A) of AZ71 in a temperature range of 25 °C to 700 °C, showing phase fractions of $10^{-4}$ to 1.

The CALPHAD calculation of the constituent phases in AZ71 over temperature ranging from fully liquid to room temperature in equilibrium state is given in Figure 12. The predominantly precipitating phase is Mg${}_{17}$Al${}_{12}$, which can nevertheless be dispersed into the solid solution by applying heat treatment temperatures over 350 °C. Hardening of AZ71 is possible via the precipitation of the Mg${}_{17}$Al${}_{12}$ phase. Additionally, a minor amount of AlMn-phases with changing stoichiometry can be found in the alloy, starting to precipitate during solidification. The Zn present in this alloy is found solely in solid solution.

Chen et al. [73] studied the forming behavior of AZ71 with rising Nd content (0–2) by rotary forging. The alloys were analyzed regarding the grain refinement properties of Nd and mechanical properties throughout the process chain: casting, homogenization, forging and annealing (T5). Suitable heat treatment parameters were shown to be a homogenization at 420 °C for 6 h and annealing at 350 °C for 1 h after forming. The rotary forging itself was done at 200 °C with 3 $s^{-1}$ up to 32% engineering strain. The alloys containing >1 Nd could be forged without surface damage. The alloy containing 1 Nd showed sufficient grain refinement in the as-cast state and the best overall tensile properties in T5 state.

In the works of Guan et al. the forming characteristics of cast AZ70 were tested [74] and a projectile head shell was produced by die forging [75]. The semi-continuous cast material was homogenized at 410 °C for 10 h to 15 h prior to forming. Flow curves, covering the range of 300 °C to 420 °C and 0.001 $s^{-1}$ to 1 $s^{-1}$ (showing DRX behavior in varying intensity), are presented in Reference [74] and were subsequently used to model the flow stress based on the ZH parameter. In Reference [75], the critical processing parameters: temperature, strain rate and degree of deformation (in relationship with the grain size) are discussed and appropriate ranges selected. Based on these a projectile head shell was forged in two steps. Forming took place with a constant crosshead speed of 8 mm$s^{-1}$ at 400 °C and 380 °C for pre-forming and punching, respectively. The finished parts were heat treated (T5 and T6) and the mechanical properties evaluated, with the T6 state showing the best overall properties.

#### 5.1.4. AZ80

The alloy AZ80 was developed in the 1950 by Dow Chemical with the specific aim to be used in forged parts, for example, die-forged wheels [13]. Even nowadays it is a commonly used alloy in the forging of Mg-products. In the as-cast state AZ80 consists of dendritic α−Mg and inter-dendritic eutectic β−Mg${}_{17}$Al${}_{12}$ phase as result of its high Al-content. To improve the forming behavior of the forging stock a homogenization heat treatment at 420 °C for 20 h was deemed suitable by Sager et al. [38]. This or a solution heat treatment makes artificially ageing (T5 or T6 states) possible for AZ80.

**Figure 13:**
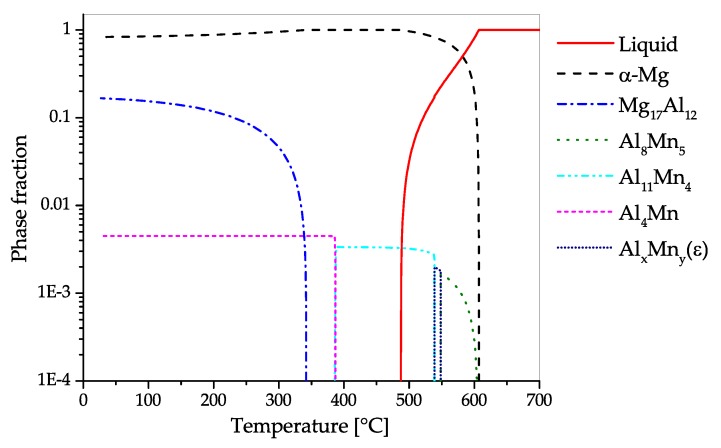
CALPHAD calculations (for further information see Appendix A) of AZ80 in a temperature range of 25 °C to 700 °C, showing phase fractions of $10^{-4}$ to 1.

The CALPHAD calculation (Figure 13), shows the fractions of phases present in AZ80 over the temperature range from fully liquid to room temperature in equilibrium state. The predominantly precipitating phase is Mg${}_{17}$Al${}_{12}$, which can dissolve into the α−Mg matrix at temperatures above 350 °C. The alloy can be strengthened by precipitation of Mg${}_{17}$Al${}_{12}$. Additionally, a minor amount of AlMn-phases with varying stoichiometry can be found in the alloy, which start to precipitate during solidification. Zinc is found solely in solid solution in AZ80.

**Figure 14:**
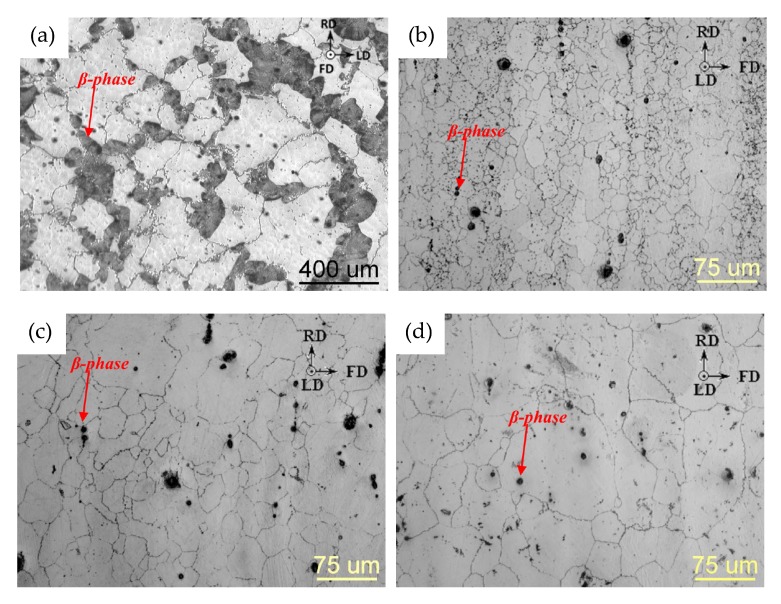
Microstructure of AZ80 in varying conditions: (**a**) cast, (**b**) forged into ‘flatbread’ samples at 350 °C with 0.65 $s^{-1}$, (**c**) forged at 450 °C with 0.65 $s^{-1}$ and (**d**) forged at 450 °C with 6.5 $s^{-1}$ [76]. Reproduced with permission from Elsevier.

The microstructure of as-cast and forged AZ80 (which is further described in Reference [76]) is depicted in Figure 14. The micrographs show the coarse casting microstructure (Figure 14a) as well as recrystallized grains resulting from the forming tests, which were performed at varying temperatures and deformation rates. Discontinuously precipitated β−particles (Mg${}_{17}$Al${}_{12}$) can be observed in the as-cast state while they are dispersed in the forged samples (Figure 14b–d). The grain size in the deformed microstructure increases visibly with increasing processing temperature and speed.

Multiple groups have investigated the material behavior of AZ80 by means of compression or upsetting tests to optimize their forging processes. Ju et al. [77] utilized such test results (0.001, 0.01 and 0.1 $s^{-1}$, up to 420 °C) for the FEM modelling of upsetting tests and an automotive wheel geometry. The simulation revealed distinct differences in strain rate and plastic strains throughout the work piece. In the work of Zhou et al. [78] processing maps in the range of 200 °C to 500 °C and 0.001 $s^{-1}$ to 20 $s^{-1}$ were generated, and the correlation between the yield strength and the Zener-Hollomon parameter shown. This approach was mirrored by Su et al. [79] who generated power dissipation maps (275 °C to 400 °C and 0.001 $s^{-1}$ to 1 $s^{-1}$) to improve the forging of aerospace cover plates from extruded stock material. The power dissipation maps showed the best processing conditions to be in a temperature range of 283 °C to 310 °C at 0.001 $s^{-1}$ to 0.0017 $s^{-1}$. This was verified by isothermal forging trails, done at 300 and 380 °C with a strain rate of 0.005 $s^{-1}$, corresponding to a ram speed of 0.075 mm$s^{-1}$. The forging produced at 300 °C exhibits a fine grain size (average of 5 μm) and good mechanical properties of 330 MPa UTS and a YS of 260 MPa, showing the best overall properties.

Kobold, Pepelnjak et al. investigated the forming behavior of extruded forging stock in multiple loading directions with compression tests and described in several works the strongly anisotropic material response. In Reference [80] the measured material parameters are used to simulate compression tests and the forging process of a shock absorber head with different FEM programs. The differences in the sample shape resulting from compression testing at 300 °C, 350 °C and 400 °C with deformation rates of 0.4 $s^{-1}$ to 2.3 $s^{-1}$ are discussed in References [81,82]. The results of these forming trials were subsequently implemented in an anisotropic yield model to successfully simulate an industrially forged part.

Similar investigations have also been conducted by Viehweger et al. [45], they studied the influence of cast versus extruded stock material on the warm forming behavior of AZ80 and AZ31. The alloys were analyzed using upsetting tests, microstructural analysis and concluding isothermal forging trials with simple testing geometries. Compression testing was done at 300, 350, 400 and 450 °C with strain rates of 0.1;1;10 $s^{-1}$, the resulting flow curves were used for a simulation of the forging process. Die forging was successfully done at various temperatures and punch speeds ranging from 1 mm$s^{-1}$ to 40 mm$s^{-1}$.

Swiostek et al. [83] compared the forging behavior of cast and extruded (350 °C, extrusion rate of 3 mmin and ratio of 8) stock material of AZ80, ZK and RE alloys by die-forging. All alloys were formed to a simple circular stepped shape in a temperature range of 200 °C to 450 °C, using die-temperatures of 220 °C. In case of AZ80 the alloy was additionally compared to two modified alloys containing 2.1 Ca and 1.9 RE respectively. The best forming temperature was found to be in a range of 350 °C to 400 °C for all alloys. The samples formed at 350 °C were further investigated by tensile, hardness and corrosion tests as well as by metallographic analysis. It was found that the modifications of AZ80 were not gainful. The microstructure of the conventional AZ80 alloy consisted of fine recrystallized grains in the range of 5 μm to 9 μm, while the modified alloys showed a coarser structure with grain diameters up to 20 μm. The unmodified AZ80 material showed superior results of all investigated AZ alloys regarding tensile strength, hardness and corrosion resistance. However, the best performing alloy throughout the study was found to be WE43.

Yoon et al. investigated the heat treatment, material behavior and process layout of three different automotive components: parts in the shape of a tie-rod, a control arm and a differential gear case. The tie-rod was forged isothermally at 250 °C from extruded stock material with a head speed of up to 560 mm$s^{-1}$ [84]. The microstructural and mechanical behavior after a T5 heat treatment (177 °C up to 55 h, reaching the peak hardness at 26 h) in comparison with the extruded stock material was of particular interest. Both, continuous and discontinuous precipitation of Mg${}_{17}$Al${}_{12}-$phase were found to occur, showing that precipitation of the continuous type takes place mainly in the fine recrystallized grains stemming from the forging process. While the strength of the extruded material was increased by approximately 50 MPa after the T5 heat treatment, the strength of the forged and heat treated AZ80 only increased by 21 MPa, whereas its elongation to failure increased from ∼6% to 8%. In Reference [85] a control arm was forged in a two-step process at material temperatures of 265 °C and 365 °C (die temperature 250 °C and ram speed of 250 mm$s^{-1}$) from extruded stock material. While the part forged at 265 °C showed the best mechanical properties, the part could not be fully formed. The mechanical properties of the part forged at 365 °C were nearly at the level of the extruded stock material while exhibiting an increased elongation to failure. The differential gear case was forged isothermally from extruded AZ80 stock material at 300 °C and a head speed of 150 mm$s^{-1}$ [86]. For an improved process design, the stock material behavior was tested in advance with compressive tests at 300 °C (1 $s^{-1}$ and 10 $s^{-1}$), these results were subsequently used to simulate the forging process by FEM. The microstructure and the mechanical properties of the as-forged parts were compared to the extruded material, showing a slight decrease in strength but an increase of elongation to failure. This behavior was attributed to DRX, leading to a decreased dislocation density and grain refinement in the forged material.

Kevorkijan et al. [87] investigated the possible use of Mg alloys in automotive applications. For this purpose, connecting rods were forged from extruded AZ80 (and ZC71/SiC/12p) at semi-industrial scale. Closed-die forming was done at a strain rate of 0.11 $s^{-1}$ using temperatures of 415 °C (material) and 300 °C (forging die). The material was heat treated (T5) at 177 °C for 24 h and the mechanical properties were evaluated. The tested part reached 389 MPa UTS and a YS of 274 MPa at an elongation of 8%.

Large helicopter support beams were forged by He et al. [88]. The filling and machine load was simulated and by choosing a slow finishing speed and a semi-open-die geometry, it was possible to forge the parts at relatively low forming loads. A pre-forged billet was used for isothermal die forging at 380 °C with speeds ranging from 1 mm$s^{-1}$ to 0.005 mm$s^{-1}$.

In the work of Wang et al. [89] the formability of cast AZ80 is analyzed by compression tests and die forging of a wheel is shown. The stock material was homogenized at 400 °C for 12 h and isothermally forged at temperatures between 360 and 400 °C using a speed of 16 mm$s^{-1}$. In the as-forged condition UTS values of 320 MPa to 330 MPa were reached.

Kurz et al. [90] compared the forming behavior of extruded AZ80 and modifications thereof (small additional amounts of Ce (<1 wt.%) as well as Ce (<1 wt.%) plus Y (<1 wt.%) to ZK60. Isothermal forgings in the shape of a stylized wheel hub were produced in a temperature range of 175 °C to 450 °C with a ram speed of 10 mm$s^{-1}$. The forgings of AZ80 showed sufficient part quality from 200 °C to 450 °C, the modification with Ce and Y had the widest forming window.

Graf et al. [48] compared the forging behavior of extruded and cast AZ31, AZ61 and AZ80—this work is discussed in Section 5.1.1.

A wheel-shaped part ( 630 mm) was produced by Yuan et al. [91] by isothermal die forging. The forming process was simulated first and based on the simulation data, forging took place at 330 °C using a speed of 1 mm$s^{-1}$. The produced parts were artificially aged at 150 °C for 30 h, before tensile tests were done at RT and 130 °C. Samples from different locations within the part were tested, which resulted in differing results of up to 100 MPa at RT. The smallest grain size and best tensile values were found in the web area; 208 MPa YS and 371 MPa UTS with an elongation of 7.5% were reached at RT. The values for 130 °C were 186;258MPa and 42.8% for YS, UTS and elongation respectively. The worse mechanical properties of the samples from the inner wall were attributed to an absence of texture, bent flow lines and a high Schmid factor, resulting in an easy activation of the basal slip.

Chen et al. [92] used isothermal closed-die forging to produce an upper receiver from extruded AZ80 in two forging steps. The forming process was simulated based on compression tests, done at 250 °C to 400 °C with 0.001 $s^{-1}$ to 1 $s^{-1}$. The forging itself took place at 16 mm$s^{-1}$ and temperatures of 320, 350 and 380 °C. Out of these temperatures, 350 °C turned out to be the most promising. The two-step process was chosen to avoid defects, for example, folds or under-fillings. The as-forged parts showed a homogeneous microstructure with a grain size of 13 μm to 18 μm throughout. In tensile tests 294 MPa YS, 406 MPa UTS and 15% $\epsilon_{f}$ were reached.

Some work in the direction of aviation applications has been done in the ‘MagForming’ project [93], where a doorstop and a blank for a compressor impeller were successfully forged from AZ80 (and WE43). Forging of the doorstop was done in two steps at 300 °C to 320 °C with a ram speed of 5 mm$s^{-1}$. The billet for the compressor wheel was forged in one step at 280 °C to 350 °C with a ram speed of 10 mm$s^{-1}$. A bigger forging, in the shape of Airbus window frame, was done at 320 °C to 330 °C using AZ80 cast plates (and AZ31 rolled plates) as stock material.

Some of the most recent work on AZ80 forged parts has been done in Canada, where parts in various sizes were produced. Forgeability of extruded and cast stock material was compared as well as the mechanical and fatigue properties investigated. The forging process itself and accompanying simulations for a control arm as well as flow curves are shown in Reference [94].

The mechanical properties of forged AZ80 were analyzed by Gryguc et al. [76,95]. Extruded and cast stock material was forged isothermally (375 °C and 20 mm$s^{-1}$ ram speed) to a part shaped like an asymmetric I-crossbeam section, and the final parts were compared. While the mechanical properties of the sample varied with texture and processing parameters, the extruded and forged material was found to be superior to the stock material in tensile tests as well as in LCF tests [95]. The same authors also investigated the mechanical behavior of as-cast and forged material (flatbread samples), which were formed isothermally in a temperature range of 350 °C to 450 °C at ram speeds of 0.65 mm$s^{-1}$ and 6.5 mm$s^{-1}$ [76]. It was found that the forging process significantly improved the properties of the cast material in the tested monotonic LCF and HCF (high cycle fatigue) regimes. The changes in microstructure originating from the forging process, refined grains, recrystallization and texture change, were stated to be the decisive factor in this regard.

The influence of the process and microstructure on the fatigue properties was also investigated by the work of Guo [96] where the spokes of an industrially forged AZ80 wheel where analyzed by tensile and fatigue testing. The microstructure was further studied with SEM and the phases α−Mg and β−Mg${}_{17}$Al${}_{12}$ were tested with nanoindentation, showing distinctive differences in stiffness. Microcracks at the boundaries between these two phases were also found to be responsible for fracture in fatigue testing.

The Mg${}_{17}$Al${}_{12}$ phases as well as the fracture behavior during the forging process was investigated as well by Qiang et al. [97]. In this work, AZ80 material was cast and homogenized at 400 °C for 15 h. Forming took place at 400 °C with a press speed of 12.4 mm$s^{-1}$. The crack initiation on the sample surface was reported to take place at the α−Mg/Mg${}_{17}$Al${}_{12}$ interface, in the sample interior cracks propagate mainly along the grain boundaries.

#### 5.1.5. AZ91

AZ91 is the most widely used Mg alloy for casting applications and it is typically processed via high pressure die casting. As a casting alloy, AZ91 benefits from its high Al amount, leading to reduced melt temperatures and high strength due to formation of intermetallic phases. Typically, high pressure cast parts made of AZ91 are used in the as-cast state.

Nevertheless, AZ91 can also be used in the wrought form, where it achieves higher strength values (as fabricated as well as T6 state) than its contenders AZ31 and AZ61, stemming from the higher Al content [98]. Despite the rather high Al content present in the alloy, the binary phase Mg${}_{17}$Al${}_{12}$, typically found on the grain boundaries, can be completely dissolved by a solution heat treatment and therefore be utilized for precipitation hardening.

The precipitation behavior has been analyzed by Braszczyńska-Malik [39], using AZ91 cast material (already mentioned in Section 5.1). After homogenization heat treatment/solution annealing (420 °C for 24 h to 26 h) the effect of artificial ageing on continuous and discontinuous precipitation was tested. It was shown that continuous precipitation is prevalent in case of dominating grain boundary diffusion while continuous precipitation was favored in case of volume diffusion. Solely continuous precipitation was achieved for a sample solution heat treated and artificially aged at 350 °C.

**Figure 15:**
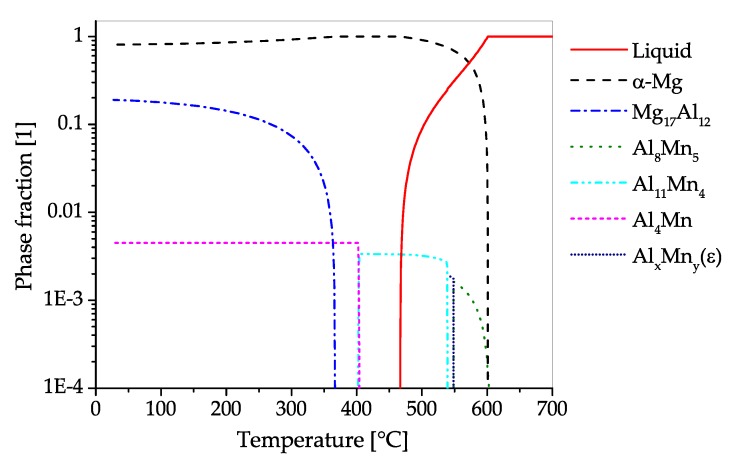
CALPHAD calculations (for further information see Appendix A) of AZ91 in a temperature range of 25 °C to 700 °C, showing phase fractions of $10^{-4}$ to 1.

The CALPHAD calculation shown in Figure 15, shows the fraction of equilibrium phases present in AZ91 over a temperature range from fully liquid to room temperature. The predominantly precipitating phase is Mg${}_{17}$Al${}_{12}$, which can also be dispersed into solid solution at annealing temperatures between 350 and 400 °C. Strengthening of AZ91 can be accomplished by precipitation of Mg${}_{17}$Al${}_{12}$. Additionally, a minor amount of AlMn-phases with varying stoichiometry can be found in the alloy, starting to precipitate during solidification. The Zn present in this alloy is found solely in solid solution.

**Figure 16:**
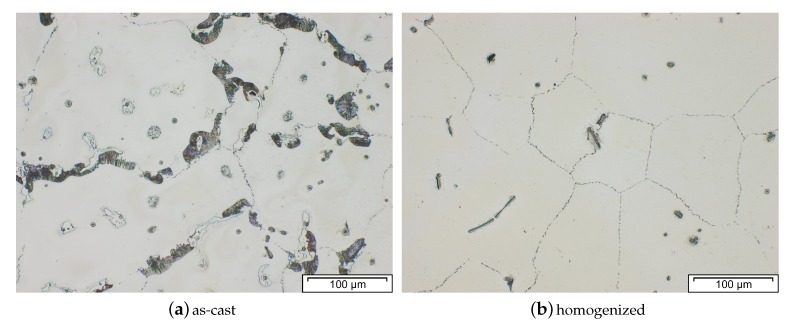

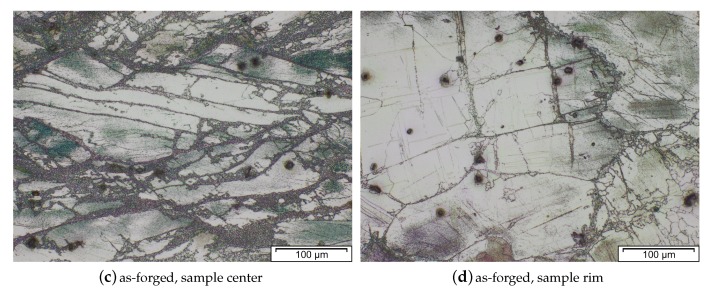
Microstructure of AZ91 in consecutive processing steps: (**a**) as-cast, (**b**) homogenized at 425 °C for 24 h. Forging took place using a material temperature of approx. 300 °C, die temperatures of 280 °C and a ram speed of 10 mms^−1^. The as-forged microstructures of parts cooled at air are shown in: (**c**) center area having high degree of deformation and (**d**) flange area with a lower degree of deformation.

The microstructure of a forged piston rod made from cast AZ91 (compare Figure 1 and Figure 4) is shown in Figure 16. Discontinuous precipitation of the Mg${}_{17}$Al${}_{12}$ phase is visible on the grain boundaries of the as-cast material (Figure a). After a homogenization heat treatment at 425 °C for 24 h, these phases are mostly dissolved into the α−Mg matrix. The homogenized material was forged at approximately 300 °C, using die temperatures of 280 °C and a ram speed of 10 mm$s^{-1}$. The as-forged parts (air-cooled) show different microstructures, depending on the degree of deformation applied. In the sample center a bimodal microstructure is observable, consisting of very fine grains, showing the onset of recrystallization and the deformed remains of the original structure. In the less deformed flange area, the cast microstructure is still visible, however, the applied deformation led to a significant amount of twins, pervading these large grains.

Owing to the fact that AZ91 is primarily a casting alloy, published works about its forging behavior are few. Madaj et al. [40] forged AZ91 at 300 °C stock temperature for a comparison with AZ31 and AZ61, this work is reviewed in Section 5.1.

The works investigating AZ91 containing Ca (AZX911) are discussed in Section 5.3.

### 5.2. Mg-Al-Mn-System

While alloys of the AM-System are not usually used for forging, the system itself has some significance for many other Al-containing alloys. Manganese is added to Mg alloys to improve the corrosion behavior by binding unwanted Fe. It can also be utilized to create dispersoids in the casting process or in subsequent heat treatment steps.

**Figure 17:**
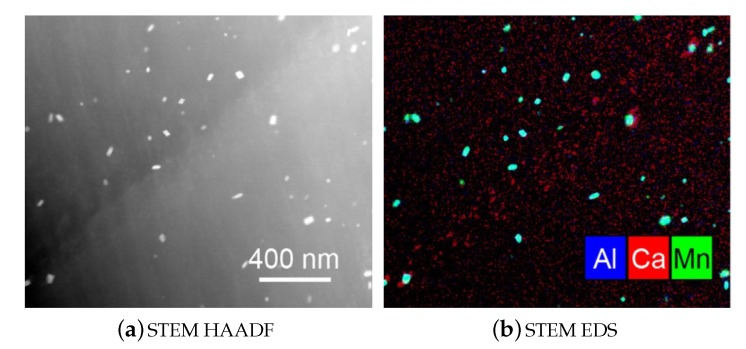
Micrographs made by scanning transmission electron microscope (STEM) high-angle annular dark-field (HAADF) (**a**) and corresponding STEM energy dispersive X-ray spectroscopy (EDS) (**b**) of Al- and Mn- containing dispersoids (blue and green, respectively) in an extruded AXM100 alloy in T6 state. Ca-containing precipitates (shown in red) are visible as well [99]. Reproduced with permission from Elsevier.

According to Cihova et al. [99] these dispersoids (Figure 17) have a negligible effect on the hardening behavior of the material, but they play an important role in the obstruction of grain boundary movement. This is of course an important feature in regard to high temperature processing and heat treatments. In their work [99], the formation and properties of these Mn-containing phases are analyzed in detail for an extruded **AXM100** alloy.

Ma et al. [100] investigated the recrystallization behavior of extruded **AM30** by compression testing followed by microstructural analyses. The isothermal testing took place at 450 °C with a strain rate of 0.001 $s^{-1}$ to 0.8 $s^{-1}$. The developed texture was analyzed by EBSD and XRD. The twinning and DRX behavior are discussed and it could be shown that the stress-strain response and the correspondent microstructure were strain rate dependent. Only at the highest strain rate (0.8 $s^{-1}$), a significant material softening during deformation was found. The recrystallization behavior changed with increasing strain rate. At higher speeds ripened, equiaxed grains evolved, whereas at low forming speeds an irregular grain structure was present, consisting of new fine recrystallized grains at the original grain boundaries besides grains growing from twin boundaries within the original grains.

### 5.3. Mg-Al-Ca-System

The addition of Ca to Mg-Al-alloys leads to various beneficial effects for wrought alloys, for example, reduced anisotropy [101,102], reduced grain size [103], increased creep resistance [104,105] and the ability of precipitation hardening by Mg${}_{2}$Ca and Al${}_{2}$Ca intermetallic phases [8]. Additionally, the oxidation tendency of Mg is reduced when Ca is present in the alloy [106,107,108], reducing possible fire hazards during alloy preparation, melt handling and high temperature processing (e.g., heat treatment, forging, …).

**Figure 18:**
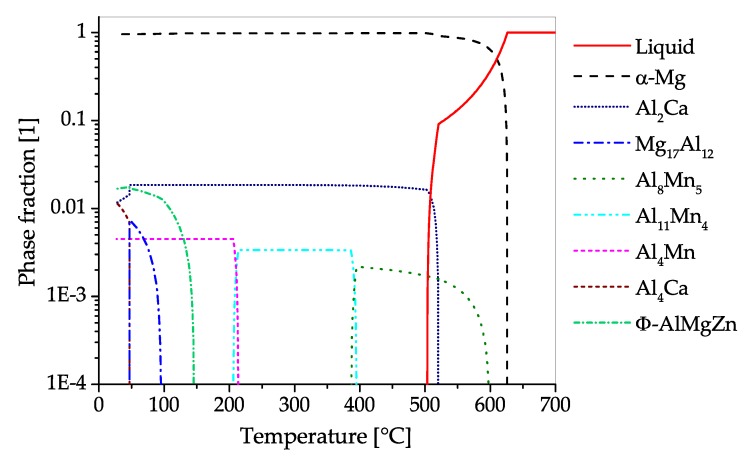
CALPHAD calculations (for further information see Appendix A) of AZX311 in a temperature range of 25 °C to 700 °C, showing phase fractions of $10^{-4}$ to 1.

The CALPHAD calculation (Figure 18), shows the fraction of phases present in AZX311 over a temperature range from the melt to room temperature in equilibrium state. If Ca is added to Mg-Al alloys either Mg${}_{2}$Ca or Al${}_{2}$Ca can be formed, this depends on the Mg-Al ratio present. In this alloy the predominant precipitating phase is Al${}_{2}$Ca, which can be used for precipitation hardening. The Al${}_{2}$Ca phase is formed while casting or in subsequent heat treatment steps. In the shown case Al${}_{2}$Ca once formed cannot be fully dispersed into the α−Mg matrix again. Additionally, a minor amount of AlMn-phases with changing stoichiometry can be found in the alloy, starting to precipitate during solidification. Zn is found mainly in solid solution, nevertheless, an AlMgZn-ternary phase can be formed at low temperatures.

**Figure 19:**
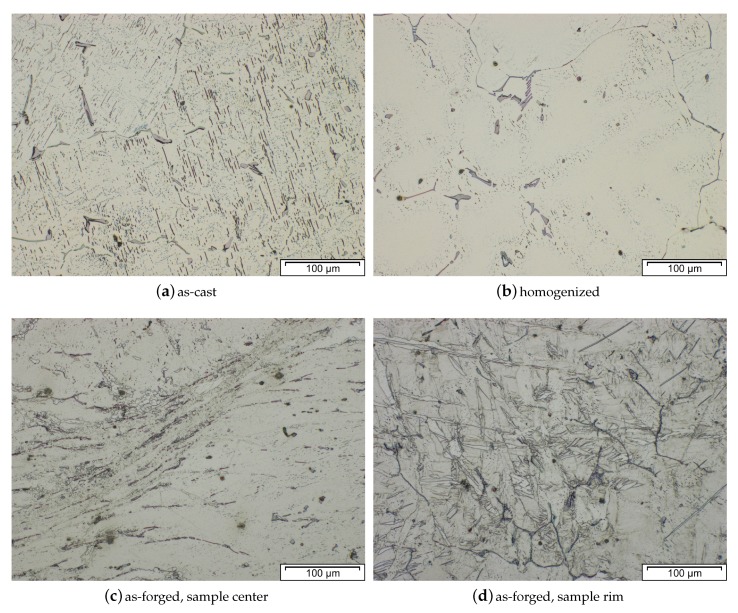
Microstructure of AZXW3110 in consecutive processing steps: (**a**) as-cast, (**b**) homogenized at 415 °C for 24 h. Forging took place using a material temperature of approx. 425 °C, die temperatures of 280 °C and a ram speed of 10 mm$s^{-1}$. The as-forged microstructures of parts quenched in water are shown in: (**c**) center area having high degree of deformation and (**d**) flange area with a lower degree of deformation.

The microstructure (cast, homogenized and as-forged) of an AZXW3110 alloy is shown in Figure 19a–d. The gravity cast material features large grains with Al${}_{2}$Ca phases precipitated at the grain boundaries and the grain interior. Most of these phases were successfully dissolved in the homogenization heat treatment, which was done at 415 °C for 24 h. From the homogenized material a piston rod (see Figure 1 and Figure 4) was forged at a ram speed of 10 mm$s^{-1}$, using temperatures of approx. 425 °C and 280 °C for material and dies, respectively, and subsequently water-cooled. The appearance of the resulting as-forged microstructure depends on the degree of deformation applied. The heavily deformed sample center shows very small recrystallized grains together with large, deformed grains. In the microstructure from the flange, were less deformation was applied, heavily twinned grains are visible, while recrystallized areas are scarce.

In the work of Kim et al. [109] AZ31 was modified with 1 Ca resulting in a **AZX311** alloy, which was electromagnetically cast and isothermally extruded at 320 °C. This stock was forged into a pulley within two forging steps. The forming process was supported by strain-rate-change tests (in the range of 200 °C to 400 °C) and a simulation of the forming steps. In the first step the billet was upset in an open die at 350 °C up to 35% engineering strain. The second step was done in a closed die at presumed temperatures of 310 °C (stock) and 320 °C (die) with a speed of 41 mm$s^{-1}$. Tensile properties were measured at various positions throughout the part, showing distinctive differences in YS, UTS and elongation. These results were explained by differences in the microstructure within the forged part.

The forging behavior of an as-cast **AZX312** alloy was investigated by Suresh et al. [110]. Therefore, compression tests were conducted in a temperature range from 300 °C to 500 °C using strain rates of 0.0003 $s^{-1}$ to 10 $s^{-1}$. These results were used to generate a processing map and to simulate a cup shaped forging. Both, the processing map and the simulation were verified by isothermal forging trials and subsequent microstructural analyses. Forging of cup shaped parts took place at 300 °C to 500 °C and 0.01 $s^{-1}$ to 10 $s^{-1}$. The recommended forging conditions, resulting in a homogeneous grain structure, are found to be 425 °C to 450 °C at a strain rate of 0.001 $s^{-1}$ to 0.01 $s^{-1}$.

A comparison of AZ31 with increasing Ca content (0–2.5) was done by Papenberg and Gneiger [32]. In this work, the Ca-containing alloys were additionally modified with ~0.25 [] Y, which is known to further improve flammability resistance. The used **AZXW** alloys were homogenized at 415 °C for 24 h after casting. Compression tests, performed at 300 °C to 400 °C with a strain rate of 0.1 $s^{-1}$ to 5 $s^{-1}$ showed an increased flow stress of the 2.5WT.%Ca containing alloy at 300 °C compared to AZ31. Closed-die forging of piston rod shaped parts was done at a stock temperature of 400 °C and a die temperature of 220 °C. The formed rods were heat treated at 350 °C for 0.5 h for recrystallization purposes. Tensile testing and microstructure analysis was done on as-forged and heat treated samples. An increasing Ca content resulted in a slightly improved YS but a decreased elongation. The heat treatment reduced the UTS and YS of the samples but improved the elongation significantly.

Hakamada et al. investigated the forming behavior of **AZX911** by means of hot compression, followed by tensile tests and microstructural analysis. In References [111,112] continuous cast AZ91 is compared to AZX911 in as-cast and homogenized (410 °C for 24 h) condition. Compression tests were conducted at 300 °C with a strain rate of 0.001 $s^{-1}$ and 0.1 $s^{-1}$ up to a true strain of 1.6. It was found that recrystallization is enhanced by the second phase particles in the Ca-containing alloy, especially if the particles are finely distributed. Also, a homogeneous distribution of Al is thought to improve the DRX properties by lowering the stacking fault energy. Therefore, the homogenized AZX911 alloy showed the most uniform grain size after compression testing, while the other tested samples exhibited a bimodal microstructure. Tensile tests at RT and 300 °C with 0.001;0.01;0.1;1 $s^{-1}$ showed an overall improvement of the tensile properties in the deformed samples when compared to as-cast material as well as large elongations to failure (284%) of the AZX911 material at 300 °C. At RT the Ca containing alloy showed an increased YS but reduced ductility in general. The influence of different grain sizes on the forming behavior of as-cast AZX911 was investigated in Reference [113] by compression tests at 250, 300 and 330 °C using 0.01;0.1;1 $s^{-1}$ up to a true strain of 1.6. A finer initial grain structure showed a slightly higher flow stress but considerably better free surface quality. An improved DRX behavior, which was found as well, is thought to originate at the smaller dispersed second phase particles (Al${}_{2}$Ca) and grain boundaries.

A die-cast **AXE622** (Mg-Al-Ca-RE) alloy was also examined by the same authors [114]. The homogenized material (410 °C for 108 h) was deformed by compression (300 °C with 0.1 $s^{-1}$ up to 1.6 true strain), followed by an analysis of the evolving microstructure and the mechanical properties. An additional sample was annealed at 425 °C for 200 h after hot deformation, which resulted in an enlarged grain structure. The compressed samples showed an increase in texture strength as well as an increased tension/compression anisotropy when compared to the as-cast material. The sample in the deformed state had the highest YS as well as compressive yield strength (YSc) of all tested samples, which was attributed to the small grain size in this condition. Annealing the deformed microstructure intensified the tension/compression anisotropy and improved the elongation in tensile testing but did not increase the strength of the material.

**Figure 20:**
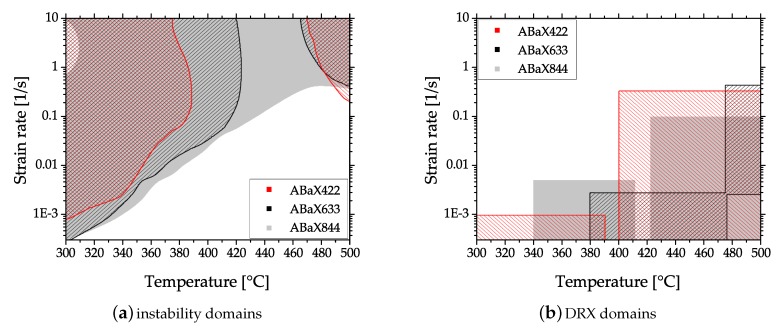
Comparison of the forming behavior at 0.5 strain of ABaX alloys with rising alloying content. Showing (**a**) domains of instability and (**b**) Dynamic recrystallization (DRX) domains, based on the processing maps calculated in References [115,116,117].

Three different **ABaX** alloys, a system interesting because of its creep resistance, were investigated by Suresh et al. [115] and Rao et al. [116,117], using a similar procedure as in References [54,110]: cup shaped parts were formed based on results of compression tests, simulation and generation of processing maps. The processing maps (calculated at a strain of 0.5) showed growing domains of instability with rising alloying content, nevertheless DRX regions, which are preferred zones for forming operations, were found as well. A comparison of the forming behavior of these alloys, based on processing maps, is shown in Figure 20. The as-cast alloys (ABaX422 [115], ABaX633 [116] and ABaX844 [117]) were formed into cup shaped parts at 300 °C to 500 °C with 0.01 mm$s^{-1}$ to 10 mm$s^{-1}$. The forming behavior of the forged samples confirmed the processing maps as the well-shaped parts were produced in the predicted DRX regimes. For these components the forming parameters varied between 380 °C and 0.01 mm$s^{-1}$ up to 500 °C with a forming speed of 1 mm$s^{-1}$. A comparison of ABaX422 and ABaX633 material [116] showed increased compressive strength (tested at 25 °C to 250 °C) and creep resistance (at 200 °C) with rising alloying content. This is thought to stem from both, an enhanced solid solution strengthening and an increased amount of intermetallic phases containing Ba and Ca.

## 6. Forging of Magnesium Alloys Containing Zinc

**Figure 21:**
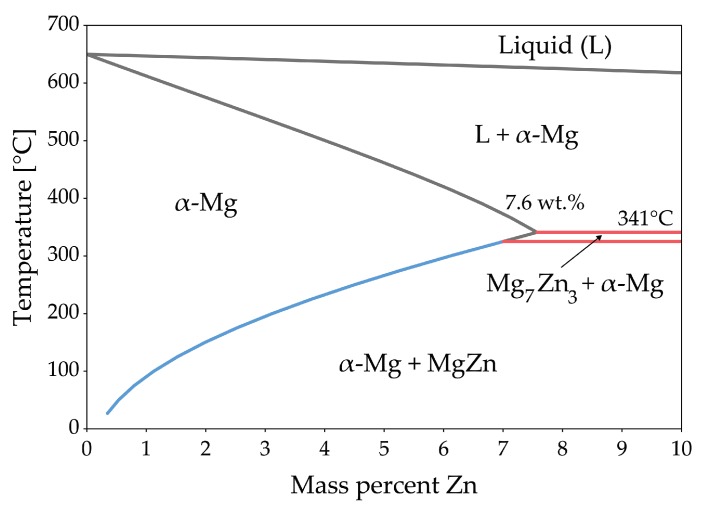
Phase diagram of the Mg-Zn system. For further information, see Appendix A.

Zinc is, besides Al, the most important alloying element for Mg, mainly due to its strengthening ability. Mg-Zn forms a eutectic phase at 340 °C which decomposes at temperatures beneath 325 °C to α-Mg and a MgZn intermetallic phase (Figure 21). It is possible to precipitation harden binary Mg-Zn alloys by utilizing coherent GP-zones as well as semi-coherent intermediate precipitates [8]. Grain refinement in Mg-Zn alloys can be achieved by the addition of Zr, leading to ZK alloys with relatively good formability and strength. Examples of alloys are ZW3 (Mg–3% Zn–>0.5%Zr), which was used as-forged aircraft wheels and helicopter gearbox housings [118], and the widely used ZK60A (Mg–6% Zn–>0.45%Zr). Because of their good biocompatibility, Mg–Zn–Ca alloys are interesting candidates for biomedical applications, which is discussed further in Section 8. Other wrought alloys using Zn as major alloying element include the Mg–Zn–Mn system, where ZM21 (Mg–2% Zn–0.5% Mn) is one well-known representative.

A **ZA21** alloy was investigated by Sanyal et al. [119] by compressive deformation. Cast stock material was cut into cuboid-shaped samples and isothermally deformed to flatbread samples between to hot plates. Forming took place at 250, 350 and 450 °C, up to a reduction of approximately 75%, subsequently the samples were quenched in water. Microstructural analysis showed a rising amount of fine DRXed grains with increasing processing temperature, nevertheless the sample deformed at 350 °C showed the least intensity of basal texture. The microstructure of all samples consisted of α−Mg and the τ−Mg${}_{32}$(Al,Zn)${}_{49}-$phase, which is typical for these type of alloys. Tensile testing showed decreasing strength with rising processing temperatures, the maximum being a YS of 232 MPa and 306 MPa UTS in the material processed at 250 °C, while the best elongation (10.7%) was found in the sample compressed at 350 °C. This decrease of material strength was attributed to the rising amount of DRXed grains and the reduction of nano-sized τ−phase particles with rising forming temperature.

### 6.1. ZK60

ZK60, containing Mg–6% Zn–>0.45%Zr, is one of the most widely known forging alloys and is applied that is, for the production of wheels in motor sport applications [3]. Because of its commercial deployment this alloy has been investigated thoroughly by various authors. The effect of twinning and stacking faults on the forming behavior and grain size of rolled ZK60 has been investigated in Reference [120]. The microstructural changes taking place during the homogenization of cast ZK60 were studied by Zhang et al. [121]. There, satisfying homogenization treatment parameters were found to be 470 °C for 14 h, resulting in an uniform microstructure without an assembly of intermetallic phases.

**Figure 22:**
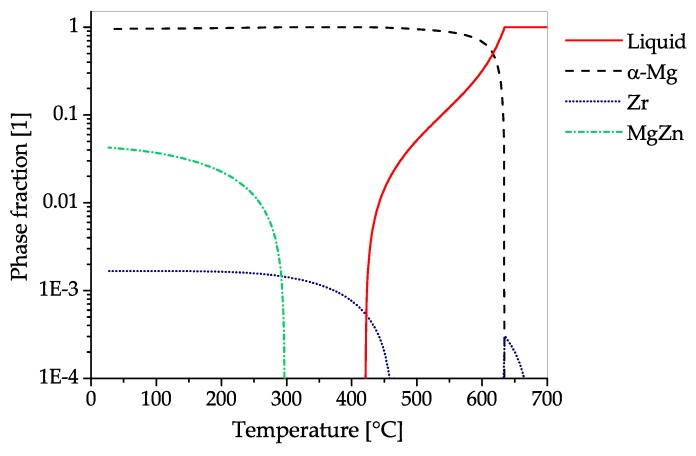
CALPHAD calculations (for further information see Appendix A) of ZK60 in a temperature range of 25 °C to 700 °C, showing phase fractions of $10^{-4}$ to 1.

The CALPHAD calculation of ZK60 (Figure 22), shows the fraction of equilibrium phases over the temperature range from fully liquid to room temperature. MgZn phases can be found in the lower temperature range, but it is possible to fully disperse these phase into the α−Mg matrix using a temperature of 300 °C to 400 °C. Additionally, a negligible amount of Zr containing phases can be found in the alloy, stemming from the Zr used for grain refinement.

**Figure 23:**
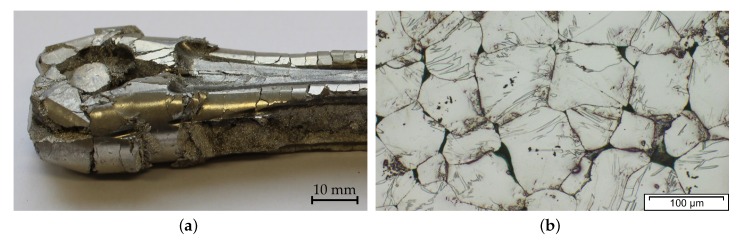
Picture (**a**) shows a piston rod, made from a ZK60 variant, ruptured in the forging process. The microstructure at the sample center is depicted in (**b**). Fracture along the grain boundaries is easily visible. A well formed part, produced from the same alloy, is shown in Figure 24.

**Figure 24:**
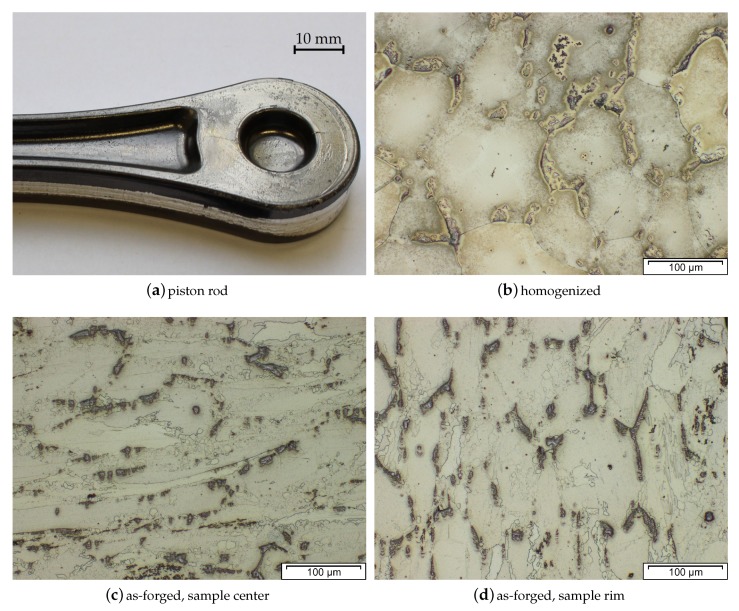
Finished part (**a**) and microstructures of a ZK60 variant in consecutive processing steps: (**b**) homogenized at 370 °C for 8 h. Forging took place using a material temperature of approx. 300 °C, die temperatures of 280 °C and a ram speed of 10 mm$s^{-1}$. The as-forged microstructures of parts cooled at air are shown in: (**c**) center area having high degree of deformation and (**d**) flange area with a lower degree of deformation.

Nevertheless, care should be taken when forging this ZK60 at high temperatures. The low-melting eutectic phases, which can be present in this alloy may cause damage of the formed part. According to Reference [30], forming at temperatures of 315 °C might cause rupturing. An example of a ruptured part and its microstructure is shown in Figure 23. The fractures along the grain boundaries of the ruptured part are easily recognizable when compared to the well-formed part shown in Figure 24.

The homogenized and as-forged microstructure of a part made from a ZKXW6000 is shown in Figure 24. This alloy is a ZK60 variant alloyed with trace amounts of Ca and Y to improve processing safety. The material was cast into a steel mold and subsequently homogenized at 370 °C for 8 h, this improves the processing behavior by a reduction of eutectic phases with a low melting temperature (MgZn). From the homogenized material a laboratory-scaled piston rod was forged at a ram speed of 10 mm$s^{-1}$. Temperatures of approximately 300 °C for the material and 280 °C for the dies were used, subsequently the formed part was cooled at air. The microstructure of the as-forged sample varies, depending on the degree of deformation applied within the part. The sample center Figure 24c shows elongated grains together with very fine grains which can be seen as the onset of dynamic recrystallization. In the lower deformed area of the flange Figure 24d, the grain structure from casting can still be recognized, also low amounts of twins and newly recrystallized grains are visible.

Processing maps for as-cast ZK60 stock (produced by squeeze casting) were created by Wang et al. [122]. Compression tests were carried out between 250 and 450 °C using strain rates of 0.001 $s^{-1}$ to 10 $s^{-1}$. The best forming condition were found to be 375 °C at a strain rate of 0.001 $s^{-1}$. The corresponding DRX domain was shown to range between 300 and 375 °C together with strain rates of 0.001 $s^{-1}$ to 0.01 $s^{-1}$. In the unstable domains of the processing map, the samples show twinning, flow localization and cracking.

These results were also used by Li et al. [123], who compared them to their own processing maps of extruded ZK60. Li et al. analyzed their material in a temperature range of 250 °C to 400 °C and 0.001 $s^{-1}$ to 1 $s^{-1}$ by compression tests. Two domains where forming should be possible without material failures were found: a DRX domain between 250 and 325 °C at strain rates of 0.001 $s^{-1}$ to 0.03 $s^{-1}$ as well as a zone, in the range of 340 °C to 380 °C and 0.001 $s^{-1}$ to 0.003 $s^{-1}$, where superplastic deformation is thought to occur. The comparison of the cast material used in Reference [122] and the extruded stock showed a distinct improvement of the forming behavior, which is attributed to the grain refinement resulting from prior extrusion.

In the work of Ogawa et al. [124] ZK60 was deformed by upsettability tests and backwards extrusion. The material was deformed with a mean strain rate of 10 $s^{-1}$ at 100 °C to 400 °C applying die temperatures of RT and 100 °C for the upper and lower die respectively. Apparently, the stock material was aged at 150 °C for 24 h before testing. The compression tests were most successful in the range of 300 °C to 400 °C. At 250 °C unstable deformation and at lower temperatures fracture was observed, over 400 °C the samples oxidized heavily. In subsequent backwards extrusion experiments cups with varying extrusion ratios were produced. There, the stock material and the container were heated to temperatures of 100 °C to 300 °C, the punch on the other hand was kept at RT. Forming was mostly successful, all forged at temperatures over 250 °C were formed without flaws, even at 200 °C cups with an extrusion ratio greater than 3.7 where produced. These results are attributed to the increased hydrostatic pressure affecting the material positively in this forming process.

A similar testing design, for both compression and backwards extrusion, is described by Matsumoto and Osakada [125]. In contrast to Reference [124], the material was brought to forging temperature directly in the heated forming tools, thereby reducing material heating and transferring time. The changes in material and processing behavior in case of billet shape modifications were investigated by simulation and forging trials. A decrease of forming load was achieved by a billet shape which allows pre-straining before the die is fully filled. The heating method showed reduced oxidation of the billets, which has its merits for small sample sizes and flat products.

Hadadzadeh et al. analyzed the forming behavior of ZK60 in as-cast and homogenized, as well as in as-extruded condition by means of compression testing [126,127]. To determine a suitable homogenization temperature, the as-cast material was tested with differential scanning calorimetry (DSC), the homogenization then took place at 400 °C for 4 h, followed by quenching in water. Isothermal compression tests were performed up to a true strain of 1 at 300 °C to 450 °C and 0.001 $s^{-1}$ to 1 $s^{-1}$. The flow curves of the extruded material show a strong anisotropy with high values in extrusion direction, as it is typical for Mg alloys. Surprisingly, the cast and homogenized samples have comparable or higher values of flow stress. Simulations based on the Ludwig equation were capable to predict the flow curves accurately and to provide insight about the strain rate sensitivity and strain hardening behavior [127]. The microstructure of the cast and homogenized material, which was analyzed in Reference [126], showed partial DRX behavior. While testing, a basal texture (perpendicular alignment of 0001${}_{Mg}$ especially present after uniaxial deformation) developed in all compressed samples. With raising temperatures and forming speed the texture intensified towards the basal poles.

Jung et al. [128] compared as-cast (die cast and semi-continuous cast) and as-extruded material by tensile testing and upsetting tests. Isothermal forming was done at 280 °C to 400 °C at 0.01;0.1 $s^{-1}$. The extruded material showed the best performance in terms of critical height reduction, followed by the semi-continuously cast samples, which reached comparable values at high forming temperatures and 0.01 $s^{-1}$. A severe drop in formability was discovered in the die-cast material above 320 °C, this behavior was attributed to low-melting eutectic phases which might cause rupturing because of the formation of micro voids.

In the works of Karparvarfard et al. [129,130,131,132] cast and extruded ZK60 was used for forging trials with various geometries accompanied by investigations of microstructure and mechanical properties. In References [129,130] cast stock was used to produce flatbread samples by isothermal open-die forging. Before deformation, the forging stock was held at forming temperature for 3.5 h. As the samples deformed with 350 °C and 0.65 mm$s^{-1}$ showed cracks after forming, only samples forged at 450 °C were used for the following investigations. Tensile and compression tests as well as analyses of texture and microstructure were conducted on a part formed from cast stock material at a speed of 0.65 mm$s^{-1}$ [129]. While the values of the forged samples for YS and UCS increased substantially (at minimum by 20 MPa), the YSc, UTS and tensile elongation did not change and the compressive fracture strain even decreased. The microstructural analysis revealed the alignment of c-axes mainly along the forging direction as well as a refined bimodal grain structure for the as-forged material. The fracture surfaces of the as-cast material showed less dimples and a more brittle appearance in general. In Reference [130] fatigue testing and fracture surface analysis were done on a sample formed at 6.5 mm$s^{-1}$. The forged samples showed higher fatigue strength when compared to the as-cast material in general. A strain dependent deformation behavior could be observed in the forged ZK60; it changed from dislocation slip to twinning and detwinning with rising strain amplitude. In the HCF regime crack nucleation is caused by persistent slip bands and intermetallic precipitates, whereas in higher strain regimes twin formation seems to be responsible for damage propagation.

A small part with varying thickness was forged from extruded ZK60 stock in Reference [131]. Semi-closed die forging was done at 450 °C and a ram speed of 0.4 mm$s^{-1}$, the produced parts were then investigated by compression testing and microstructural analysis. The forged compression samples showed a slight reduction of strength but an improvement of failure strain when compared to the as-extruded material. This behavior is mirrored on the fracture surface, where dimples—a sign for increased plastic deformation—can be found mainly on the forged samples. This behavior is attributed to the changes in microstructure: the bimodal structure from the as-extruded material changed to a pancake structure, accompanied by an increase of texture intensity with rising degree of deformation in the formed part.

As-cast ZK60 was used as stock material for a larger forging in the shape of an I-beam section in Reference [132]. Isothermal closed-die forging was done at 250 °C with a speed of 20 mm$s^{-1}$. The formed part showed a bimodal grain size, consisting of elongated grains surrounded by small round grains, which indicates DRX. Furthermore, a basal texture started to develop in the forged material, based on the degree of deformation. In the subsequent tensile and fatigue testing a distinct improvement of the forged samples was shown when compared to the as-cast material. The YS (depending on the sample location) was increased by up to 93% (268 MPa) but the elongation reduced to 9% $\epsilon_{f}$. The fatigue life, tested stress controlled at 140, 160 and 180MPa using R=−1, showed an order of magnitude improvement in the forged material, regardless of the loading stress. This is attributed to the grain refinement and texture of the forged part, resulting in a higher strength and an alteration of the deformation mechanisms.

Simulations of the forged parts analyzed by Karparvarfard at al. in Reference [132], as well as the forging process of an automotive control arm made of extruded ZK60 are described in the work of Paracha [94], which has already been mentioned in Section 5.1.4.

An investigation of the forging behavior of extruded AZ31 and ZK60 by Poerschke [52] has already been discussed in the Section 5.1.1.

A comparison of forgings made from extruded stock material, published by Swiostek et al. [83] was already described in Section 5.1.4. Amongst others, ZK60 was compared to a modified ZK60 alloy with additional 2.1 RE and to a ZK30 alloy. The samples produced by die forging at 350 °C (F-temper) showed an inhomogeneous microstructure, consisting of large deformed grains as well as fine recrystallized ones. The tensile properties of all ZK alloys were similar, with ZK60 reaching the highest UTS but the RE modified alloy showed the best YS with the lowest elongation. ZK30 was reported to exhibit the best corrosion resistance among the ZK alloys, nevertheless all other tested materials (various AZ80 alloys and WE43) had substantially lower corrosion rates than the ZK alloys.

Kurz et al. [90] analyzed the forming window of extruded ZK60 and modifications thereof (small additional amounts of Ce (<1 wt.%) as well as Ce (<1 wt.%) plus Y (<1 wt.%), in comparison to AZ80. In the forming trials (described in the Section 5.1.4), the modification of ZK60 with Ce improved the forming behavior at low (175 °C) and high temperatures (up to 450 °C). The unmodified ZK60 alloy on the other hand failed by hot cracking at a forming temperature of 400 °C.

## 7. Forging of Magnesium Alloys Containing Rare Earth Elements

Rare earth elements (REE or RE) are a group of seventeen metals including the lanthanides, Y and Sc, which can often be found together in geological deposits. The group can further be divided into ‘light’ rare earth elements (lanthanides La to Eu—atomic numbers 57 to 63) and ‘heavy’ rare earth elements (Y, Sc and lanthanides from Gd to Lu—atomic numbers 64 to 71). Although it is postulated that most properties of REE are rather similar [133], there are significant differences when alloyed to Mg. In common, all of them form eutectics with Mg and exhibit a certain amount of solid solubility. High-temperature intermetallic compounds, that can form with Mg are often used to generate creep-resistant Mg alloys [134] and can also be utilized for precipitation hardening [8]. Wrought Mg-alloys containing REE offer a wide variety of alloying systems ranging from industrial established Mg-Zn-RE [13,135] and Mg-Y-RE alloys (e.g., WE43) to high strength Mg-REE alloys with LPSO (long period stacking order) structure [136,137].

### 7.1. WE43

WE43 is considered a benchmark alloy for a good combination of mechanical properties, corrosion and oxidation behavior. It has been investigated intensively and is used in various applications. Moreover, it is a candidate of interest for for example, the aviation industry. Since a relatively high amount of expensive Y is used, this alloy is higher priced than most other Mg alloys. On the other hand, Mg-RE alloys using Ce and La or Mischmetal (Ce/La) are comparably cheap.

**Figure 25:**
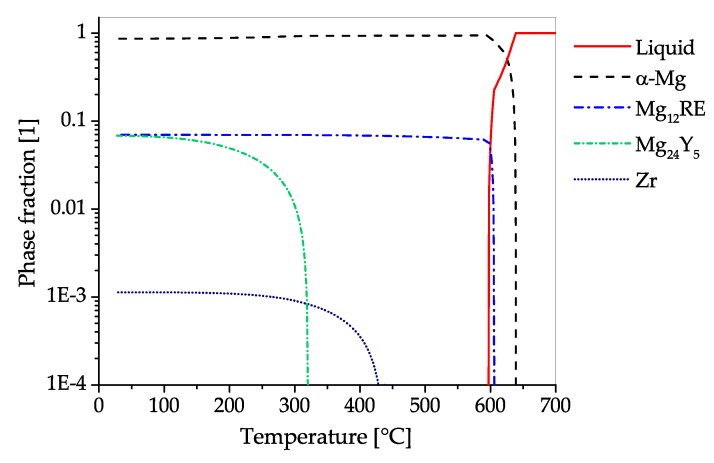
CALPHAD calculations (for further information see Appendix A) of WE43 in a temperature range of 25 °C to 700 °C, showing phase fractions of E−4 to 1.

The CALPHAD calculation (Figure 25) shows the fraction of phases present in WE43 over the temperature range from fully liquid to room temperature in equilibrium state. High-temperature stable Mg-RE phases already start to form in the melt or may precipitate in subsequent heat treatments. In contrast, the Mg${}_{24}$Y${}_{5}$ phase is only stable up to approximately 320 °C. In case of ageing treatments this Y-containing phase or not yet precipitated RE phases can be used for precipitation hardening. Small amounts of predominantly Zr-containing particles may also occur in this alloy.

**Figure 26:**
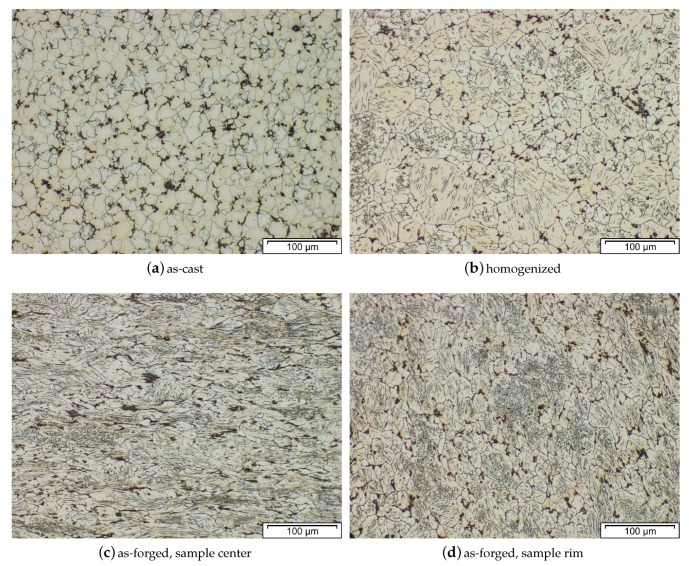
Microstructure of WE43 in (**a**) as-cast state as well as (**b**) homogenized at 525 °C for 24 h. The material was forged at temperatures of 380 °C and 280 °C for material and die respectively using a ram speed of 10 mm$s^{-1}$. The as-forged, air-cooled microstructures of (**c**) sample center and (**d**) sample rim are depicted as well.

The microstructure (cast, homogenized and as-forged) of a WE43 alloy is shown in Figure 26. The as-cast microstructure (Figure 26a) consists of grains with an average size >50 μm and intermetallic phases mainly located along the grain boundaries. The cast material was then homogenized at 525 °C for 24 h (Figure 26b) in order to improve the processing behavior via a reduction of phases at the grain boundaries. The grain size increased visibly, also newly precipitated RE-phases can be seen in the grain interiors. From the homogenized material a piston rod (see Figure 1 and Figure 4) was forged at a ram speed of 10 mm$s^{-1}$, using temperatures of approximately 380 °C and 280 °C for material and dies respectively. The formed part was air-cooled. The as-forged microstructures of sample center and sample rim look similar. The grain shapes and size are reduced corresponding to the applied degree of deformation, but the structure itself did not change considerably from the homogenized material, showing phases on the grain boundaries and grain interior.

Panigrahi et al. [33] investigated the ageing behavior of upset forged WE43. The forging stock (cube shaped samples cut from a WE43 plate in F temper) were forged at 380 °C and 1 $s^{-1}$ up to a deformation of 1.2 true strain. The effect of varying T5 ageing temperatures (150, 180 and 210°C) was investigated by hardness measurements, tensile testing and metallographic analysis which included EBSD measurements and an investigation of the fracture surface. The highest tensile strength (344 ± 11 and 388 ± 12 MPa, for YS and UTS respectively) was reached by the sample heat treated at 180 °C for 60 h. These high values are thought to stem from overlapping effects of work hardening, fine grain size and precipitation strengthening.

In the comparison of forging alloys presented by Swiostek et al. [83] (already discussed in Section 5.1.4 and Section 6.1), as-extruded WE43 stock was forged at ranging from 450 °C to 300 °C, using a die temperature of 220 °C. The parts formed from WE43 developed cold cracks at forging temperatures of 300 °C and required the highest forging forces of all compared materials (various ZK and AZ80 alloys). The forming force of WE43 showed a distinctive drop between 350 °C and 400 °C, nearly dropping to the level of the other alloys. As-forged samples from all alloys deformed at 350 °C were further investigated. The microstructure of the analyzed sample from WE43 showed a fine-grained microstructure with grain diameters of 5 μm to 9 μm. WE43 also displayed the highest values for YS, UTS, elongation and hardness at room temperature, as well as the best corrosion resistance in salt spray testing.

Henry et al. [138] used extruded forging stock to investigate the formability and mechanical properties of open-die forged WE43. The extruded stock material was heat treated at 525 °C for 8 h before forming. Cylindrical samples were compressed to a ‘pancake’ shape using temperatures of 360 °C to 480 °C with strain rates of 0.006 $s^{-1}$ to 8 $s^{-1}$ to reach deformations of 35%–85% strain. The microstructure of the forged parts varied widely. Heavily deformed grains with coarse precipitates (360 °C) could be found as well as fully recrystallized grains without visible precipitates (480 °C), dependent on the forming speed. A T5 heat treatment (recommended at 180 °C to 260 °C) was used to reach a YS >225 MPa and UTS of 350 MPa.

Some work on the topic of aviation applications has been done in the ‘MagForming’ project, where a door stop and a blank for a compressor impeller were successfully forged from WE43 (and AZ80) [93]. Especially for the compressor wheel the good mechanical properties of WE43 at high temperatures are a topic of interest. Forging of the door stop was done in two steps at 300 °C to 350 °C with a ram speed of 5 mm$s^{-1}$. The billet for the compressor wheel was forged in one step at temperatures between 300 °C and 400 °C with ram speeds of 10 mm$s^{-1}$ to 30 mm$s^{-1}$.

### 7.2. Alloys with LPSO Structure

This type of alloy was produced the first time by rapidly solidified powder metallurgy in the early 2000 s. Soon afterwards it was shown that casting was also a viable route of production [139]. These alloys, mainly of the Mg–Zn–Y and Mg–Zn–Gd systems, combine high strength and acceptable room temperature ductility with good high temperature performance. A detailed overview of the microstructure and phases as well as the most relevant works for these alloys is given in References [136,137].

Mg-Zn–Y alloys do not only form the LPSO-phase (Mg${}_{12}$ZnY), but may as well contain varying amounts of α−Mg, MgZn, I-phase (Mg${}_{3}$YZn${}_{6}$), W-phase (Mg${}_{3}$Y${}_{2}$Zn${}_{3}$) and β−Mg (Mg${}_{24}$Y${}_{5}$). The preferred phase is shown to be closely dependent on the Zn/Y ratio present in the alloy, and follows the forming trend of:

α−Mg→β−Mg (eutectic)→ LPSO−phase → W−phase (dendritic) → I−phase (quasicrystal)

with rising Zn/Y ratio [136]. The LPSO-phase itself has several possible stacking sequences (i.e., 6H, 10H, 14H, 18R, 24R structure type) which may change during processing steps and heat treatment.

As the production of relevant quantities of this alloys by casting was made possible, forming (and forging) soon became a topic of interest. Nevertheless, the works about forgings still seem to be limited to basic characterizations with upset forging being the main forming process. For a better overview of the investigated alloys discussed in this section, the composition ( wt.% and at.%) of each are listed in Table 3.

Asakawa and Hirukawa [140] investigated the forming behavior of the alloys **Mg-2Gd-1Zn** and Mg-2Gd-1Zn-0.2Zr with upset forging. The as-cast material was heat treated at 520 °C for 2 h, quenched in water with 80 °C and annealed again at 400 °C for 1 h. Forming took place at 380 °C with a press speed of 5 and 250 mm$s^{-1}$ up to reduction ratios of 30, 50 and 80%. The variation of cooling rate while casting and the addition of 0.2 Zr reduced the grain size considerably (from 2800 μm to 57 μm) and positively influenced the forming behavior. This material also showed favorable tensile properties of 299 MPa UTS and an elongation of 15.4%. The influence of forging temperature, speed and degree of deformation on forming behavior and mechanical properties of the parts was investigated as well. While higher temperatures and a low forming speed show improved quality of the formed part, the mechanical properties were enhanced with reduced temperatures and low forming speed but with increased reduction ratio.

In the works of Han, Xu and Shan [141,142] a cast **GWZK102** alloy was forged and aged. The homogenized (510 °C for 10 h and quenched in water with 80 °C) forging stock was formed at 470 °C, subsequently the material was aged at 200, 225 and 250°C for up to 80 h. While an effect of age hardening could be shown for all samples, the highest strength was reached after 60 h at 200 °C, reaching values of 406 MPa UTS with 5.9% elongation. The in-depth study of the microstructure showed a grain refinement of the α−Mg grains and precipitation of the LPSO phase (14H type) during forging. These LPSO phases are known to inhibit dislocation glide and grain growth. Moreover, the formation of $\beta^{\prime }-$phase precipitates during the ageing process was found. The high strength of the GWZK102 alloy is therefore attributed to these precipitated LPSO and $\beta^{\prime }-$phases. The overageing visible in this alloy is thought to stem from precipitate free zones, grain growth and coarsening of secondary phases.

The same alloy was forged to the shape of a bracket in a study presented by Shan et al. [143]. The forging process was simulated in advance and the die design was adapted according to the results. The material was forged isothermally at 407 °C (at presumably 1 mm$s^{-1}$) to two differing billet shapes. The parts were successfully produced and subsequently aged at 200 °C for up to 80 h. The peak strength was reached after 63 h, showing tensile properties of 243 MPa YS, 380 MPa UTS and an elongation of 4.07%. The increase in strength is attributed to the precipitation of $\beta^{\prime }$ and $\beta^{\prime \prime }-$phases, but LPSO phases were not mentioned specifically.

**Figure 27:**
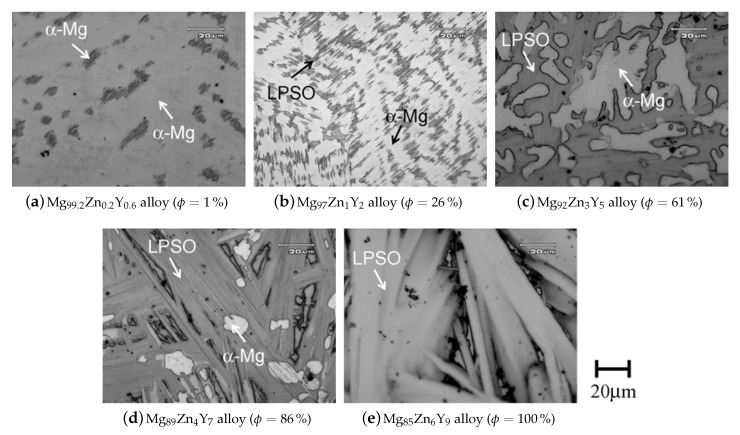
Microstructure of as-cast Mg-Zn-Y alloys with varying long period stacking order (LPSO) volume fraction (ϕ) [144]. The scale bar applies to all figures (**a**–**e**). Reproduced with permission from Elsevier.

A comparison of **Mg-Zn-Y** two-phase alloys with different LPSO phase fractions was done by Matsumoto et al. [144] using cast material with varying amounts of Zn (6, 4, 3, 1 and 0.2) and Y (9, 7, 5, 2 and 0.6). Therefore, the approximate volume fraction (ϕ) of the LPSO Phase ranged from 1%–100%, changing the material behavior accordingly. Figure 27 shows the as-cast microstructures of the investigated alloys. The cast material was homogenized at 500 °C for 10 h before doing upsettability tests at material temperatures in a range of 200 °C to 500 °C and an initial strain rate of 0.31 $s^{-1}$ (8.3 mm$s^{-1}$). Isothermal testing was performed at 200 °C, for all other temperatures the tool was heated to 250 °C. While all alloys showed comparable behavior at 500 °C the alloys Mg${}_{92}$Zn${}_{3}$Y${}_{5}$ and Mg${}_{89}$Zn${}_{4}$Y${}_{7}$ showed high strength even at higher temperatures. Values of up to 400 MPa were reached at 200 and 300 °C. This performance is thought to be related to the interaction of the α−Mg and LPSO phase boundaries. Isothermal forging experiments (setup comparable to Reference [59]), were done on the Mg${}_{97}$Zn${}_{1}$Y${}_{2}$ alloy at 300 °C with an average speed of 80 mm$s^{-1}$. A simulation of the forging process and the forces required, based on the rule of mixture (α−Mg and LPSO phase), was implemented and showed improved results when compared to conventional methods.

Matsumoto et al. compared cast with extruded Mg${}_{97}$Zn${}_{1}$Y${}_{2}$ by compression experiments in an earlier publication [145]. Testing was done as already described before, and while the extruded samples showed a high strength at 200 and 300 °C, the ductility was not improved when compared to the cast material. This behavior changed at higher temperatures for the material extruded at 350 °C which showed good formability at 400 °C testing temperature. While the α−Mg phase accommodated deformation by twinning, the LPSO structures showed kink deformation. Both results are to be expected in these types of alloys.

Two **Mg–Zn–Y–Zr** alloys containing the eutectic I-phase were investigated by Garcés et al. [146]. The alloys, containing Zn${}_{2.34}$Y${}_{0.29}$Zr${}_{0.11}$ and Zn${}_{4.92}$Y${}_{0.58}$Zr${}_{0.12}$ (in at.%), were compressed to a reduction of 92%. Three passes were applied, in which the material was reheated to the starting temperature of 400 °C and held at this temperature for 20 min after each pass. In the subsequent tensile and compression tests at RT, values of ~350 MPa UTS and UCS >450 MPa were reached by the higher alloyed material. After forming, fracturing of the I-phase (contributes to grain refinement) and the precipitation of Mg-Zn binary phases (known for strength improvement) are shown by TEM analyses.

## 8. Forging of Biodegradable Magnesium Alloys

While biodegradable products made from Mg alloys are commercially available nowadays, forging is not a common production process. The possible alloying elements in biodegradable alloys are greatly restricted and product dimensions and pricing make uncommon production methods that is, ECAP viable. Nevertheless, a short introduction to biodegradable Mg alloys is subsequently given and works using forged material discussed.

Mg is a trace element, naturally present in the human body and also reported to be beneficial for bone healing [147]. Therefore, Mg alloys are in the focus as fully biodegradable implant materials for screws, plates, pins and stents. A full consumption of tissue-supporting implants during and after rebuilding of the damaged bone or tissues eliminates the necessity of a second surgical operation for implant removal. Major requirements are excellent biocompatibility of all alloying elements and a very low risk of allergic reactions. Accordingly, Mg-Zn-Ca (ZX) alloys are attractive candidates for biodegradable implants. A sufficiently low in-vivo degradation rate is required to ensure the stabilization during the healing process (up to 12 weeks). Further, the formation of gaseous H${}_{2}$, which usually goes along with implant degradation, has to be controlled to amounts tolerable by the surrounding tissue. The degradation rate is influenced by the difference in the chemical potential of matrix and precipitates [148] and also by the volume fraction of intermetallic precipitates, as well as grain size [149] and matrix composition [150,151]. Moreover, it is recommended to keep the content of the common impurities of Mg as Be, Fe, Ni and Cu as low as possible, because they tend to precipitate and form corrosion cells in Mg-alloys [152].

**Figure 28:**
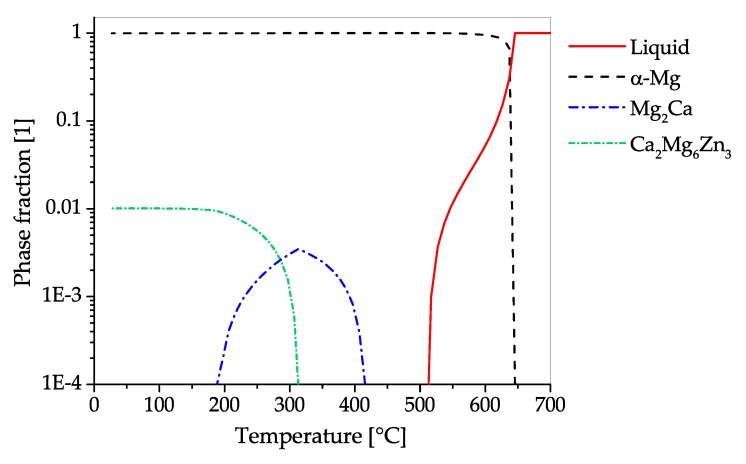
CALPHAD calculations (for further information see Appendix A) of ZX10 in a temperature range of 25 °C to 700 °C, showing phase fractions of $10^{-4}$ to 1.

The CALPHAD calculation (Figure 28), shows the fraction of present phases in ZX10 over the temperature range from fully liquid to room temperature in equilibrium state. During casting only the α−Mg is forming. Because of the low content of alloying elements the volume content of precipitating phases is very low. Nevertheless, Mg${}_{2}$Ca may form between 200 °C and 400 °C. A ternary phase, Ca${}_{2}$Mg${}_{6}$Zn${}_{3}$, can precipitate between RT and 300 °C.

With respect to biocompatibility, the applicable alloying elements are strictly limited. There exist numerous studies concerning the strength-enhancing effects of rare earths, Zr, Y, Zn and Ca, but in particular Zn and Ca eliminate the risks concerning insufficient biocompatibility. As intermetallic particles or rare earths might accumulate in the human body, lean Mg-Zn-Ca alloys are of great interest.

Calcium is a major constituent of hydroxyapatite Ca${}_{5}$(PO${}_{4}$)${}_{3}$(OH), the crystalline phase of bone. It is reported to improve the osseointegration rate and to be beneficial for bone healing, however, for biocompatibility, the alloy should contain max. 1 of Ca [153]. Intermetallic phases in Ca-containing Mg alloys are reported to distribute uniformly and to be advantageous for processing and final microstructures, as they facilitate particle stimulated nucleation and DRX [154]. During degradation of Mg-Ca alloys, Ca is consumed by the surrounding bone. If Ca is in solid solution, the corrosion rate is slightly reduced [155], whereas the influence of intermetallic Ca-containing precipitates on the corrosion behavior strongly depends on the respective chemical potential of matrix and precipitates [156].

Alloying Mg with Zn leads to a strength increase by solid-solution (systematically analyzed in Reference [157] and calculated to amount about 20 MPa per wt.%). The other possible strengthening mechanism is the formation of intermetallic precipitates, for example, Ca${}_{3}$Mg${}_{6}$Zn${}_{3}$ in combination with Ca or—if the Zn content is reduced—(Mg,Zn)${}_{2}$Ca, which is less noble than the Mg-matrix [158] and diminishes therefore selective corrosion attack. Zinc is also reported to enhance DRX during hot-working [159].

Besides the requirement for excellent biocompatibility, also adequacy of mechanical properties for the application in biodegradable implants has to be ensured. The elastic modulus of Mg alloys of 41–45 GPa is closer to that of bone (3–20 GPa) than the elastic modulus of Ti-, Fe- or Zn-based alloys, which reduces the risk of bone-matter decomposition, strength loss and stress-shielding [148]. The initially low yield strength (YS) of <30 MPa and ductility of as-cast Mg can be enhanced significantly by grain refinement, solid solution strengthening and age hardening.

Intermetallic particles, which are present during hot working are reported to enable particle stimulated nucleation and, subsequently, DRX. This is shown by Zhang et al. for Mg-Zn-Ca alloys [154] or by Robson et al. for Mg-Mn alloys [160]. Their grain boundary pinning effect promotes a fine-grained microstructure after hot-working and/or annealing [150,161]. Grain refinement is a very effective method of strengthening in Mg-alloys, as the strengthening coefficient is more than four times higher than in Al-alloys [162]. Besides grain-boundary strengthening, a further positive effect of a fine-grained microstructure, resulting from DRX, is the weakening of a strong basal texture. This results in a reduction of the directional strength-anisotropy of the final implant and the risk of failure of the implant.

At present, the main application for hot and cold working of Mg-alloys is not to produce the final shape of an implant, but to take advantage of a wide range of possibilities for microstructure adjustment and design. The standard production process for biodegradable Mg-alloys usually comprises a homogenization treatment, followed by extrusion, forging or hot rolling and an optional annealing and/or a further deformation process as hot- or cold-working or severe plastic deformation (SPD). As the development of biodegradable Mg-alloy tends to a limitation of alloying elements to nutritional elements and a reduction in the content of alloying elements, the following description of several studies comprising hot and cold working follows this trend in a similar order.

The relationships of processing and properties of the alloy system ZX, representing Mg-Zn-Ca alloys, were investigated in several works. Kang et al. present, in Reference [163], a study of the influence of the point of the homogenization annealing within a two-step deformation process of a Mg –4% Zn –0.5%Ca (**ZX41**) alloy. The initially conducted homogenization heat treatment comprises a first annealing step at 380 °C for 20 h and a second holding step at 510 °C for 3 h. The forging was performed at 300 °C and a speed of 0.1 $s^{-1}$ to a height reduction of 50% and extrusion was performed at 280 °C with an extrusion ratio of 16:1 and a ram speed of 0.01 mm$s^{-1}$. Conducting the homogenization heat treatment after the forging process lead to dissolution of the intermetallic MgZn${}_{2}$ particles at the grain boundaries. The authors concluded that this hindered DRX during the following extrusion process and, therefore, resulted in increased YS and ultimate tensile strengths (UTS) of 320.7 MPa and 385.2 MPa, respectively, together with an elongation of 12.8%. The reduced fraction of DRX is further reported to maintain a pronounced basal texture, which additionally contributed to the increase in strength. If the homogenization heat treatment was conducted before the forging step, a high amount of stored energy led to a higher recrystallized fraction during and after subsequent extrusion. The lower YS and UTS of 271 MPa and 370.9 MPa as well as the higher elongation of 21.7% are explained by the almost fully DRXed microstructure.

The influence of processing parameters on microstructure and mechanical properties of a Mg–1% Zn–0.3% Ca (**ZX10**) and Mg–0.5% Zn–0.15% Ca (**ZX00**) alloy and, moreover, the influence of intermetallic particles present during deformation were studied by Hofstetter et al. [164]. The cast material was initially homogenized and solution annealed at 350 °C for 12 h and 450 °C for 8 h. Prior to hot forming, different heat treatments were conducted at 250 °C, in order to homogeneously distribute the intermetallic particles in the ZX10 and to dissolve them in the ZX00 material. The hot forming behavior of the material was analyzed by compression tests in a deformation dilatometer at 325 °C to 450 °C and finally by direct and indirect extrusion tests. In both extrusion tests an extrusion rate of 25:1 and a ram speed of 0.5 $s^{-1}$, corresponding to a strain rate of 0.74 $s^{-1}$, were applied. The authors show that a high volume fraction of Laves phase particles during hot-deformation leads to a fine grained microstructure, but the amount of particles has only a slight effect on the recrystallized volume fraction. A comparison between direct and indirect extrusion shows higher volume fractions of recrystallized grains (97% compared to 78%) at an extrusion temperature of 325 °C. Fully recrystallized microstructures could be obtained by a final heat treatment, which might lead to severe grain growth in the particle-free ZX00 alloy. The best-balanced mechanical properties (238 MPa YS, 265 MPa UTS, 31% $\epsilon_{f}$) were achieved after indirect extrusion at 300 °C. The ZX10 material, produced from Mg in ultra-high purity and processed with the same extrusion parameters (except a reduced ram speed of 0.15 $s^{-1}$) shows an excellent *in-vivo* degradation performance, which was assigned to the very low content of impurities and intermetallic particles [150]. Grün et al. already tested ZX00 material (aged and indirectly extruded at 300 °C) in small and large animals, showing low degradation rates, adequate H${}_{2}$ evolution and undisturbed bone formation and in-growth [165].

How forging speed and temperature of an open-die forming process influence the microstructure, mechanical properties and finally the corrosion resistance of a **binary Mg–1% Ca alloy** is presented by Harandi et al. in Reference [156]. A variation of the preheating temperature from 250 °C to 350 °C and 450 °C showed that increasing forging temperatures lead to refined grains in the final material, resulting in higher ductility, whereas the maximum hardness was achieved at a forging temperature of 350 °C. In contrast, the variation of the forging speed of 40, 50, 60 and 65 stocks per minute did not result in significant changes. Mg${}_{2}$Ca particles were found inside the α-Mg grains as well as at the grain boundaries, but the overall phase fraction decreased with increasing forging temperature, which is a possible explanation for the reduction of hardness in the material forged at 450 °C. The corrosion resistance of the forged material could not be improved compared to the as-cast material.

As some studies show that the degradation rate is partially influenced by the grain size, processing Mg-alloys by SPD can be a promising way for refining and homogenizing the microstructure [166]. The most practicable SPD processes are equal channel angular pressing and high-pressure torsion as they allow to process sufficiently large amount of material for mechanical testing as well as implant manufacturing.

## 9. Forging of Various Magnesium Alloying Systems

In the work of Zheng et al. [167], the flange of an automotive clutch was forged of as-cast **NZ30K** (Mg–Nd–Zn–Zr), an alloy which is known for good creep resistance. The forming conditions were determined in tensile (200 °C to 400 °C, at 0.01 $s^{-1}$) and compression tests (250 °C to 400 °C, at 0.001 $s^{-1}$ to 10 $s^{-1}$) primarily showing the temperature dependence of the forging process. The best results were expected between 350 °C to 400 °C with a strain rate of 0.001 $s^{-1}$ to 1 $s^{-1}$. Therefore, forming took place at 400 °C stock temperature and 250 °C die temperature, followed by artificial ageing (T5). The mechanical properties were investigated by tensile testing, the T5 state showing YS and UTS values of 318 MPa and 324 MPa respectively. Creep testing was done at 200 and 250 °C with an applied stress of 100 MPa for up to 150 h. The measured steady-state creep rate of NZ30K at 200 °C is an order of magnitude lower than the creep rate of AZ91, confirming the good high temperature behavior of NZ30K.

An investigation of multiple **TAZ** (Mg–Sn–Al–Zn) alloys regarding their forging behavior, grain size evolution and mechanical properties was done by Yoon and Park [71]. They compared the performance of extruded forging stock made from TAZ541, TAZ711 and TAZ811 to AZ61 and AZ80. The TAZ alloys were homogenized at 460, 480 and 500°C for 12 h, the AZ stock was homogenized at 440 °C for 15 h. The parts were forged in a temperature range of 250 °C to 450 °C with the strain rates of 2 $s^{-1}$ to 10 $s^{-1}$. A T-shaped part was used to analyze the forging behavior of the alloys, while a part in the approximate shape of a control arm was used for further testing. In the forming process the TAZ alloys showed better performance when compared to the AZ samples. The analysis of the parts forged at 450 °C showed a bimodal microstructure in the TAZ711 alloy, while the grain size of the AZ61 part was quite homogeneous. The tensile properties of the as-forged samples depend on the processing temperature, the forgings at 250 °C reached the highest strengths in this study. The alloys TAZ711 and TAZ811 show a higher YS but a reduced elongation when compared to the AZ samples. Tested TAZ alloys reached YS of 360 MPa and UTS of 380 MPa.

Semi-closed die forging of a **TX31** alloy was done by Rao et al. [168], using as-cast stock material. TX31 shows a good creep resistance due to ternary Mg-Sn-Ca phases in the Mg matrix. In their work Rao et al. analyze the forming behavior of TX31 by means of a processing map (at a strain of 0.5), that shows two domains of interest for forming. The experimental trials, where cylindrical stock was forged into cup shaped parts, were done at 350 °C to 500 °C using a forming speed of 0.01 mm$s^{-1}$ to 10 mm$s^{-1}$. The forging experiments were accompanied by simulations, which allowed to describe the forces necessary for forming accordingly. The microstructure of the forged parts varied in terms of grain size as well as shape and size of the secondary particles. The samples forged in the domain ranging from 350 °C to 500 °C at strain rates of 1 $s^{-1}$ to 10 $s^{-1}$ showed preferable properties. The microstructure consists of DRX grains and a reduced amount of Mg-Sn-Ca particles, the forming parameters are therefore recommended by the authors for the use in forging operations.

## 10. Concluding Remarks

Before concluding this work, it might be beneficial to look at the conclusions drawn and prospects given at the dawn of Mg production, therefore three quotes from a review authored by Haughton in 1939 are discussed briefly in the following:

*The reactive nature of magnesium has seriously interfered with its much wider adoption; to those who still associate the metal mainly with the ribbon which is burned to give a flashlight for photography, it seems incredible that magnesium can be used for structural purposes* [22].

While not many people still know that Mg once was used for photography flashlights, this statement is in principle still valid today. A big issue to this day is the fire hazard conceived by industry and consumers, showing itself in the ban of Mg products in aircraft interiors until 2015 [169] by the Federal Aviation Administration (FAA) or the brochure on ‘Safe Magnesium’ issued by the International Magnesium Association (IMA) [170]. This ultimately lead to the development of ‘non flammable’ Mg alloys either containing high amounts of RE elements (e.g., WE43) or Ca (and Y) which reduce the oxidation tendency and partially even exhibit self-extinguishing behavior.

Another topic linked to the reactive nature of Mg and its alloys is the corrosion behavior, an important aspect for producers and users of Mg products. This directly correlates to the next quote:

*Nothing has been found or appears likely to be found, which will produce a “stainless magnesium”.* [22]

The corrosion behavior of Mg has been an issue since the beginning of its use in structural applications. While various methods for corrosion protection exist [7] and have been known since the first half of the 20th century [22], so called ‘stainless’ Mg alloys have only been reported in 2015 by Xu et al. [171]. In their work a Mg-Li alloy was shown to form a stable Li${}_{2}$CO${}_{3}$ film with complete coverage on the surface, protecting the Mg-Li matrix beneath from corrosion.

The final quote from 1939 highlights another main nuisance for the production and use of Mg alloys (i.e., wrought alloys), the poor ductility at RT, that has already been a hot topic to that time:

*Much more progress has been made on alloys for use at room temperature. … Work is now being carried out at the laboratory on the properties of alloys which have been rolled at 200 °C. This work is not yet complete, but interesting results have already been obtained* [22].

The ductility shown by Mg alloys is of course dominated by their hexagonal crystal structure, which does only provide 3 of the necessary 5 independent slip systems for arbitrary deformation at RT. The activation of additional slip systems above 200 °C is therefore important for any major forming operation.

The urge to improve the deformation behavior of Mg alloys at low temperatures is prevalent in many activities, showing itself notably in the development works on sheets, where the use of RE and Ca containing alloys has shown to improve the ductility by texture modification. Also, the utilization of fine grain sizes, leading to a better distribution of deformation throughout the microstructure, is a topic in this regard.

The recently published (2018, 2019) works of Wu et al. [172] and Ahmad et al. [173], in which a modulation of the hexagonal crystal structure by alloying is discussed, gives rise to new expectations in terms of low temperature ductility of Mg alloys in the near future.

While a lot of issues hindering industrial application have been solved and the development as well as understanding of these alloys has increased over the years, there is still potential to improve processability and performance in use.

Some topics of interest for Mg forgings have been discussed by Suh et al. [174] in their review on Mg sheet alloys, that is, texture development and age hardenability.

The anisotropy generated by forming is an issue for all Mg forming processes. Especially sheet and extruded products show distinctive textures [175] which can have profound influences on the mechanical material properties. The use of extruded stock material for forged products influences therefore the forging process itself as well as the properties of the final product [82]. The texture solely created by forging on the other hand is markedly reduced, when compared to extrusion and rolling, and might therefore not be such an intensely discussed topic.

The development of age hardenable alloys on the other hand is an important future topic for forged products as well. The absence of studies discussing such parts is easily visible throughout this review. While some authors conduct T5 treatments [33,91,138] and others have shown the increase of material ductility after recrystallization treatment [32,75], there are nearly no forged parts in T6 state mentioned in the investigated literature [75,99]. This might be the case because only few Mg alloys exist which show age hardening to a strength beyond that gained by work hardening in the forging process.

A comparatively new type of alloying design for Mg alloys aims to change this. These materials called ‘micro alloyed’ [176] or ‘lean alloy’ [99] try to achieve a high final strength by fine grain sizes and precipitation hardening, utilizing heat treatments after the forming processes. In the forming process itself these alloys show low strength, permitting forming operations with reduced forces. A further benefit is the reduction of material costs by using low amounts of alloying elements.

Alloys which aim for application in higher temperature regimes are still heavily dependent on the use of RE alloying elements. The development of the materials showing LPSO phases is an intriguing development in this regard and it will be interesting to see the progress made with these alloys in the future.

The recycling of Mg products is not a topic in this work, nevertheless a few words should be said, as it plays an important role in the overall structure of the Mg market. The raw materials for primary production of magnesium are available in unlimited abundance but its extraction is very energy intensive. Recycling of already existing alloys is much more energy-efficient and therefore of vested industrial interest. While some products are recycled in grade and can therefore be used repeatedly for Mg products, several specific problems like rapid melt oxidation and complex removal of impurities hinders efficient recycling in general. Additionally, a vast amount of magnesium is dissipated in use (i.e., for iron desulphurization, Grignard reagents and pyrotechnics) or used as an alloying element for aluminum alloys and therefore lost for recovering metallic magnesium. An overview on the topic of magnesium recycling can be found in the work of Ditze and Scharf [177], additional in-depth information on the recycling of Mg in the EU (2017) is given in Reference [178].

While the increasing knowledge and development as well as the possible future research topics of Mg forgings have been described in this section, one main issue—the low rate of industrial application—has not yet been discussed. According to the review authored by Sillekens et al. [11], the main reason for this is the sub-critical market size for wrought Mg products, which can be described as a feedback loop: the lack of available stock material [83] promotes a scarceness of know-how, which accordingly decreases the possible applications and increases the prices of realized products, which in turn dampens the industrial interest.

Mg forgings are predominantly made from deformed feedstock (i.e., extruded or rolled), in order to take advantage of the already refined grain size for the forging operations. Thereby, the improved material flow behavior aids the die filling and is beneficial for the surface quality of the produced part. This stock material is needed in a wide range of shapes and sizes for various forgings. While it is possible to buy extruded material and plates, the market is small and the variety of available alloys is even smaller. The use of cast forging stock gives more freedom of choice in case of alloy selection and reduces the price by skipping one forming step but has disadvantages as well. Depending on the applied casting process and part size, the grain size and number of defects may increase, also machining/scalping steps will most likely be necessary.

The know-how necessary for industrial-sized forging applications is diversified. Production parameters like die design, process layout, heat treatments and machining behavior have to be known, but also additional technologies as for example surface treatments need to be available. After production the resulting part properties have to be verified. This commonly includes corrosion tests and mechanical testing to obtain for example, tensile properties and for some parts fatigue or creep testing as well as fracture mechanical investigations might be required. For many of these listed points even basic information is hardly available and the appropriate development effort can be intense.

The above listed problems but also the already mentioned conceived fire hazard and susceptibility of Mg alloys to corrosion are inhibiting factors for a broad range of industrial applications. The parts available at present are mainly found in the high-priced fields like defense and sportive products. In these cases the whole process chain can be trimmed to optimal performance of one product, but this mostly entails protected process design and small product quantities, which does little to increase the overall industrial acceptance. Nevertheless, the ever-increasing range of Mg alloys, better mechanical properties and improved understanding of production routes rise hope for an increased amount of Mg wrought products in the coming years.

In particular, we expect an increase of industrial forged parts made from Mg alloys in the near future, as supported by market trends, such as lightweighting. The wide range of possible processing parameters for forgings, when compared to other Mg forming processes, eases the entry into the production of Mg forgings for manufacturers. Further progress might be achieved by the use of alloys designed for specific forming operations, thereby improving the processing behavior, increasing work safety and enhancing the properties of the finished part.

The authors hope that this work, which aims to give an overview of the processing parameters and possible resulting properties of Mg forgings, can provide useful aid in the future production of such components.

## Figures and Tables

**Table 1:** Common alloying element designations based on the ASTM Standard Alloy Designation System.

Element			Element		
Designation	Name	Abbrev.	Designation	Name	Abbrev.
A	aluminum	Al	N	nickel	Ni
B	bismuth	Bi	P	lead	Pb
Ba	barium	Ba	Q	silver	Ag
C	copper	Cu	R	chromium	Cr
D	cadmium	Cd	S	silicon	Si
E	rare earth	RE/REE	T	tin	Sn
F	iron	Fe	V	gadolinium	Gd
H	thorium	Th	W	yttrium	Y
J	strontium	Sr	X	calcium	Ca
K	zirconium	Zr	Y	antimony	Sb
L	lithium	Li	Z	zinc	Zn
M	manganese	Mn			

**Table 2:** Nominal chemical compositions of commercial Mg-Al-Zn alloys given in wt.%.

Alloy	Al	Zn	Mn	Si	Cu	Fe	Ni	Others	Mg
AZ31 [36]	2.5–3.5	0.5–1.5	0.05–0.4	<0.1	<0.1	<0.03	0.005	0.1 max	Balance
AZ61 [36]	5.5–7.0	0.5–1.5	0.15–0.4	<0.1	<0.1	<0.03	0.005	0.1 max	Balance
AZ80 [36]	7.8–9.2	0.2–0.8	0.12–0.3	<0.1	<0.05	<0.005	-	0.3 max	Balance
AZ91A [37]	8.5–9.5	0.45–0.9	>0.17	<0.05	<0.025	<0.004	>0.001	0.01 max	Balance

**Table 3:** Overview of the chemical compositions of the investigated alloys in the works reviewed in Section 7.2 given in wt.% and at.%.

Alloy	Y		Gd		Zn		Zr		Mg	Source
	wt.%	at.%	wt.%	at.%	wt.%	at.%	wt.%	at.%		
Mg-2Gd-1Zn			11.5	2.00	2.40	1.00			Balance	[140]
Mg-2Gd-1Zn-0.2Zr			11.4	2.00	2.38	1.00	0.66	0.20	Balance	[140]
GWZK102	2.00	0.61	10.0	1.73	0.50	0.21	0.30	0.09	Balance	[141,142,143]
Mg-Zn-Y	2.15	0.60			0.53	0.20			Balance	[144]
	6.84	2.00			2.51	1.00			Balance	[144,145]
	15.5	5.00			6.82	3.00			Balance	[144]
	20.4	7.00			8.58	4.00			Balance	[144]
	24.6	9.00			12.4	6.00			Balance	[144]
Mg-Zn-Y-Zr	1.01	0.29			5.99	2.34	0.39	0.11	Balance	[146]
	1.93	0.58			12.0	4.92	0.41	0.12	Balance	[146]

**Figure A1:**
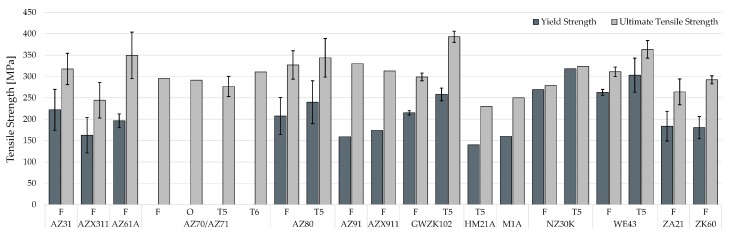
Tensile properties of various forged Mg alloys with differing tempers, tested at RT. The graphic shows mean values calculated from various scientific sources, the error bars show standard deviation. The used sources are listed in Table A5.

## Abbreviations

The following abbreviations and symbols are used in this manuscript:

DRX	dynamic recrystallization	SPD	severe plastic deformation
DSC	differential scanning calorimetry	STEM	scanning transmission electron microscope
EBSD	electron backscatter diffraction	TEM	transmission electron microscope
ECAP	equal channel angular pressing	UCS	ultimate compressive strength
EDS	energy dispersive X-ray spectroscopy	UTS	ultimate tensile strength
FAA	Federal Aviation Administration	XRD	X-ray diffraction
FEM	finite element method	YS	tensile yield strength
HAADF	high-angle annular dark field	YSc	compressive yield strength
HCF	high cycle fatigue	ZH	Zener-Hollomon parameter
HPT	high pressure torsion		
IMA	International Magnesium Association	ϕ	volume fraction
LCF	low cycle fatigue	$\epsilon_{f}$	elongation to failure
LPSO	long period stacking order	$\epsilon_{p}$	elongation at peak
RE/REE	rare earth elements	$\epsilon_{other}$	undefined elongation
RT	room temperature	at.%	atomic percent
SEM	scanning electron microscope	wt.%	weight percent

## Appendix A. CALPHAD Calculations

The figures showing binary phase diagrams and CALPHAD calculations were done with the software Thermo-Calc Version 2019a. For this purpose the databases Thermo-Calc TCBIN v 1.1 and ThermoTech TTMG5 were used, respectively. The specific alloy compositions used are shown in Table A1.

**Table A1:** Alloy compositions in wt.% used for CALPHAD calculations in this work.

Alloy	Al	Zn	Mn	Ca	Zr	Y	Ce	La	Mg
AZ31	3.0	1.0	0.2						Balance
AZ61	6.3	1.0	0.2						Balance
AZ71	7.69	0.54	0.18						Balance
AZ80	8.0	0.5	0.2						Balance
AZ91	9.0	0.625	0.2						Balance
AZX311	3.0	1.0	0.2	1.0					Balance
WE43					0.4	4.0	1.74	1.16	Balance
ZK60		6.0			0.6				Balance
ZX10		1.0		0.3					Balance

## Appendix B. Heat Treatments and Forming Temperatures

A short overview of common temper designations used in Mg alloys is given in Table A2; Table A3 summarizes recommendations for forging and die temperatures for various Mg alloys. Additionally, a selection of commonly used temperatures and times for Mg heat treatments is given in Table A4.

**Table A2:** Common temper designations based on Reference [179].

Designation	Explanation
F	as-fabricated
O	annealed, recrystallized
W	solution heat treated
T4	solution heat treated and naturally aged
T5	artificially aged only
T6	solution heat treated and artificially aged
H1	strain hardened only
H2	strain hardened and partially annealed
H3	strain hardened and stabilized

**Table A3:** Recommended forging temperatures for various Mg alloys in °C by Reference [30].

Alloy	Workpiece	Forging Die
AZ31B	290–345	260–315
AZ61A	315–370	290–345
AZ80A	290–400	205–290
EK31A	370–480	345–400
HM21A	400–525	370–425
QE22A	345–385	315–370
ZE42A	290–370	300–345
ZE62	300–345	300–345
ZK21A	300–370	260–315
ZK60A	290–385	205–290

**Table A4:** Heat treatment parameters of various Mg alloys, based on recommendations from literature.

	Alloy	Parameter	Source
**Homogenization**			
	AZ31	450 °C for 3 h	[66]
	AZ70	410 °C for 10 h to 15 h	[75]
	AZ71	420 °C for 6 h	[73]
	AZ80	420 °C for 20 h	[38]
		400 °C for 12 h	[89]
	AZ91	413 °C, 16 h to 24 h	[35]
		413 °C, 6 h + 352 °C, 2 h + 413 °C, 10 h	[35]
	ZK60	470 °C, 14 h	[121]
	ZK61	499 °C, 2 h	[35]
		482 °C, 10 h	[35]
	WE43	525 °C, 4 h to 8 h	[35]
**Anneal/ stress relieving**			
	AZ31 O	345 °C, 2 h	[35]
	AZ31 F	260 °C, 15 min	[35]
	AZ31 H24	150 °C, 1 h	[35]
	AZ61 F	260 °C, 15 min	[35]
	AZ80 F	260 °C, 15 min	[35]
	AZ80 T5	200 °C, 1 h	[35]
	ZK60 F	260 °C, 15 min	[35]
	ZK60 T5	150 °C, 1 h	[35]
**T5 − artificial ageing**			
	AZ71	350 °C, 1 h	[73]
	AZ80	177 °C, 16 h to 24 h	[35]
	AZ91	216 °C, 4 h	[35]
		168 °C, 16 h	[35]
	ZK60	150 °C, 24 h	[35]
	ZK61	149 °C, 48 h	[35]
	WE43	180 °C, 60 h	[33]
**T6 - solution heat treatment ${}^{†}$ + artificial ageing**			
	AZ91	413 °C + 168 °C, 16 h	[35]
		413 °C + 216 °C, 5 h to 6 h	[35]
	ZK61	499 °C + 180 °C, 16 h	[35]
	WE43	525 °C, quenching in water at 65 °C + 250 °C, 16 h	[35]
**Maximum Temperatures**			
	AZ91	418 °C	[35]
	ZK61	502 °C	[35]
	WE43	535 °C	[35]

†: Times for solution heat treatment are usually process and part dependent and can therefore not be given here.

## Appendix C. Mechanical Properties

In this section the mechanical properties of various Mg alloys are listed. The values are directly taken from the sources given and might concern forged samples as well as stock material. The processing parameters, where available, are listed as well.

The mechanical properties of forged samples tested at room temperature are given in Table A5. The properties of stock material and forged samples at elevated temperatures are listed in Table A6. A graphical overview of the mechanical properties at room temperature of various forged Mg alloys is given in the Figure 5 and Figure A1, using the values listed in Table A5.

**Table A5:** Mechanical properties of various forged Mg alloys tested at room temperature.

Alloy	Temper	YS	UTS	$\epsilon_{f}$	$\epsilon_{\mathrm{other}}$	YSc	UCS	Source	Process Parameters
		[MPa]	[MPa]	[%]	[%]	[MPa]	[MPa]		
AZ31		170	260		15 (in 50 mm)			[27]	
	F	290	360		15 (A5)			[58]	rolled + forged at 410 °C, temperature ranging of 250 °C at 0.6 mm$s^{-1}$, sample from rib position
	F	221	312		12 (A5)			[58]	rolled + forged at 410 °C, temperature ranging of 250 °C at 0.6 mm$s^{-1}$, sample from base position
	F	220–298	310–365		11–16 (A5)			[56]	rolled and annealed plate, forged at a stock temperature of 410 °C and a die temperature of 200 °C at 0.6 mm$s^{-1}$, one-rib bracket
	F	216–250	303–350		12–15.5 (A5)			[56]	rolled and annealed plate, forged at a stock temperature of 410 °C and a die temperature of 200 °C at 0.6 mm$s^{-1}$, two-rib bracket
	F	136	278	12.4		78–116	278–316	[66]	cast, homogenized at 450 °C for 3 h, isothermal forged at 450 °C and 6.5 mm$s^{-1}$
	F	180–189	347–369					[49]	extruded + formed at 300 °C to 400 °C, 12 mm$s^{-1}$ to 160 mm$s^{-1}$ using a die temperature of 350 °C
	F	234	280		14.4 (A5)			[64]	forged at 400 °C material and die temperatures of 300 °C in three steps, using a screw press
AZX311	F	121–204	203–286		21–41			[109]	cast+extruded 320 °C + two step forged, the billet was upset in an open die at 350 °C up to 35% engineering strain; second step in a closed die at presumed temperatures of 310 °C (stock) and 320 °C (die) with 41 mm$s^{-1}$
AZ61	F	180	295		12 (in 50 mm)	125		[27]	
	F	194–231	398–410	18–21				[49]	extruded + formed at 300 °C to 400 °C, 12 mm$s^{-1}$ to 160 mm$s^{-1}$ using a die temperature of 350 °C
AZ70	F		295.2	8.88				[75]	cast + homogenized at 410 °C for 10 h to 15 h, two step forged with 8 mm$s^{-1}$ at 400 °C and 380 °C respectively
	O		291.5	11.52				[75]	cast + homogenized at 410 °C for 10 h to 15 h, two step forged with 8 mm$s^{-1}$ at 400 °C and 380 °C respectively
	T5		300.2	9.12				[75]	cast + homogenized at 410 °C for 10 h to 15 h, two step forged with 8 mm$s^{-1}$ at 400 °C and 380 °C respectively
	T6		310.7	13.28				[75]	cast + homogenized at 410 °C for 10 h to 15 h, two step forged with 8 mm$s^{-1}$ at 400 °C and 380 °C respectively
AZ71	T5		253		10.7			[73]	AZ71 + 1 Nd, cast + homogenized at 420 °C for 6 h, rotary forged at 200 °C with 3 $s^{-1}$ to an eng. strain of 32%, annealed to T5 at 350 °C for 1 h
AZ80	F	230	330		11 (in 50 mm)	170		[27]	
	T5	250	345		6 (in 50 mm)	195		[27]	
	F	173.8	311.9	14.1		124.5	373	[76]	cast + isothermal forged at 350 °C, 0.65 mm$s^{-1}$
	F	176.9	310.6	15.8		111.2	366.7	[76]	cast + isothermal forged at 450 °C, 0.65 mm$s^{-1}$
	F	181.7	306.1	14.2		69.2	325.9	[76]	cast + isothermal forged at 450 °C, 6.5 mm$s^{-1}$
	F	110.7	239.8	7.2				[95]	cast + isothermal forged at 375 °C, 20 mm$s^{-1}$
	F	175	312.1	14.1				[95]	cast + isothermal forged at 375 °C, 20 mm$s^{-1}$
	F	226.8	351.1	17.5				[95]	extruded + isothermal forged at 375 °C, 20 mm$s^{-1}$
	F	219.2	341.3	20.5				[95]	extruded + isothermal forged at 375 °C, 20 mm$s^{-1}$
	F	196	315	16		159	402	[86]	
	F	206	318					[79]	extruded + isothermal forged at 380 °C, 0.075 mm$s^{-1}$
	F	260	330					[79]	extruded + isothermal forged at 300 °C, 0.075 mm$s^{-1}$
	F	248	361	15		190		[85]	extruded + forged at 265 °C, die temperature 250 °C, 20 mm$s^{-1}$
	F	207	349	16		175		[85]	extruded + forged at 365 °C, die temperature 250 °C, 20 mm$s^{-1}$
	T5	274	389		1			[87]	extruded + forged at 400 °C, die temperature 300 °C, 0.11 $s^{-1}$, heat treated (T5) at 177 °C for 24 h
	F		320-330					[89]	cast + homogenized at 400 °C for 12 h and isothermal forged at 360 °C to 400 °C and 16 mm$s^{-1}$
	T5	280.2	370.6		7.5			[91]	forged at 330 °C with 1 mm$s^{-1}$, artificially aged at 150 °C for 30 h, sample in web position
	T5	155.5	270.3		5.3			[91]	forged at 330 °C with 1 mm$s^{-1}$, artificially aged at 150 °C for 30 h, sample in flange
	F	294	406	15				[92]	extruded + forged with 16 mm$s^{-1}$ at 350 °C
AZ91	F	159	330	16.1				[111]	cast + homogenized (410 °C for 24 h), compressed at 300 °C with 0.1 $s^{-1}$ up to 1.6 true strain
AZX911	F	174	313	10.7				[111]	cast + homogenized (410 °C for 24 h), compressed at 300 °C with 0.1 $s^{-1}$ up to 1.6 true strain
GWZK102	F	210	308		4.2			[141]	cast + homogenized at 510 °C for 10 h and quenched in water with 80 °C, forged at 470 °C
	T5	273	406		5.9			[141]	cast + homogenized at 510 °C for 10 h, quenched in water with 80 °C, forged at 470 °C, ageing at 200 °C for 60 h
	F	220	290		5.94			[143]	isothermal forged at 407 °C at presumably 1 mm$s^{-1}$
	T5	243	380		3.84			[143]	isothermal forged at 407 °C at presumably 1 mm$s^{-1}$, T5 ageing at 200 °C for 63 h
HM21A	T5	140	230		15 (in 50 mm)	115		[27]	
M1A		160	250		7 (in 50 mm)			[27]	
NZ30K	F	269.3	278.8		12.2			[167]	cast + forged at 400 °C stock temperature and 250 °C die temperature, with a strain rate of 0.001 $s^{-1}$ to 1 $s^{-1}$
	T5	318.2	323.5		11.2			[167]	cast + forged at 400 °C stock temperature and 250 °C die temperature, with a strain rate of 0.001 $s^{-1}$ to 1 $s^{-1}$
WE43	T5	> 225	350					[138]	extruded + heat treated (525 °C for 8 h), forged (360 °C to 480 °C) with strain rates of 0.006 $s^{-1}$ to 8 $s^{-1}$ to an amount of 35%–85% strain, aged at 180 °C to 260 °C
	F	263 ± 7	311 ± 11		23 ± 3			[33]	F temper plate, forged at 380 °C and 1 $s^{-1}$ up to a deformation of 1.2 true strain
	T5	286 ± 10	341 ± 4		28 ± 1			[33]	F temper plate, forged at 380 °C and 1 $s^{-1}$ up to a deformation of 1.2 true strain, ageing at 150 °C for 104 h
	T5	344 ± 11	388 ± 12		23 ± 1			[33]	F temper plate, forged at 380 °C and 1 $s^{-1}$ up to a deformation of 1.2 true strain, ageing at 180 °C for 60 h
	T5	318 ± 9	368 ± 10		17 ± 1			[33]	F temper plate, forged at 380 °C and 1 $s^{-1}$ up to a deformation of 1.2 true strain, ageing at 210 °C for 32 h
ZA21	F	232 ± 4	306 ± 11		7.8 ± 0.4			[119]	cast + isothermally forged at 250 °C, quenched in water
	F	168 ± 2	250 ± 2		10.7 ± 1.2			[119]	cast + isothermally forged at 350 °C, quenched in water
	F	151 ± 7	236 ± 7		7.1 ± 1.1			[119]	cast + isothermally forged at 450 °C, quenched in water
ZK60		215	305		16	160		[27]	
	F	163 ± 10	286 ± 4	26 ± 3		111 ± 1	390 ± 6	[129]	cast + forged at 450 °C, 0.65 mm$s^{-1}$

**Table A6:** Mechanical properties of Mg alloys tested at elevated temperatures.

Alloy	Temper	Temperature	Testing Speed	UTS	YS	$\epsilon_{p}$	$\epsilon_{f}$	$\epsilon_{\mathrm{other}}$	YSc	Source	Process Parameters
		[°C]	[$s^{-1}$]	[MPa]	[MPa]	[%]	[%]	[%]	[MPa]		
AZ31	as-cast	300	1						139	[48]	
	as-cast	300	5						140	[48]	
	as-cast	300	10						148	[48]	
	as-cast	350	1						113	[48]	
	as-cast	350	5						117	[48]	
	as-cast	350	10						122	[48]	
	as-cast	400	1						88	[48]	
	as-cast	400	5						95	[48]	
	as-cast	400	10						104	[48]	
	extruded	300	1						166	[48]	
	extruded	300	5						189	[48]	
	extruded	300	10						230	[48]	
	extruded	350	1						120	[48]	
	extruded	350	5						150	[48]	
	extruded	350	10						171	[48]	
	extruded	400	1						74	[48]	
	extruded	400	5						100	[48]	
	extruded	400	10						113	[48]	
AZ61	as-cast	300	1						134	[48]	
	as-cast	300	5						145	[48]	
	as-cast	300	10						150	[48]	
	as-cast	350	1						105	[48]	
	as-cast	350	5						122	[48]	
	as-cast	350	10						127	[48]	
	as-cast	400	1						90	[48]	
	as-cast	400	5						101	[48]	
	as-cast	400	10						108	[48]	
	extruded	300	1						160	[48]	
	extruded	300	5						200	[48]	
	extruded	300	10						210	[48]	
	extruded	350	1						125	[48]	
	extruded	350	5						150	[48]	
	extruded	350	10						170	[48]	
	extruded	400	1						75	[48]	
	extruded	400	5						100	[48]	
	extruded	400	10						120	[48]	
AZ80	as-cast	325	1.5d-4	42.3	38.6	1.5	6.2			[38]	cast
	cast + hom	325	1.5d-4	45.5	42.5	1	13			[38]	cast + hom. at 420 °C, 5 h
	cast + hom	325	1.5d-4	46.7	43.5	2.5	10.1			[38]	cast + hom. at 420 °C, 20 h
	as-cast	375	1.5d-4	24.6	24.4	0.5	14			[38]	cast
	cast + hom	375	1.5d-4	28.4	27.8	0.75	10.8			[38]	cast + hom. at 420 °C, 5 h
	cast + hom	375	1.5d-4	26.4	24.4	0.6	17.3			[38]	cast + hom. at 420 °C, 20 h
	as-cast	420	1.5d-4	14.6	14.3	0.65	2.1			[38]	cast
	cast + hom	420	1.5d-4	15.7	15.1	0.75	17.1			[38]	cast + hom. at 420 °C, 5 h
	as-cast	300	1						148	[48]	
	as-cast	300	5						160	[48]	
	as-cast	300	10						171	[48]	
	as-cast	350	1						113	[48]	
	as-cast	350	5						128	[48]	
	as-cast	350	10						139	[48]	
	as-cast	400	1						92	[48]	
	as-cast	400	5						108	[48]	
	as-cast	400	10						114	[48]	
	extruded	300	1						149	[48]	
	extruded	300	5						214	[48]	
	extruded	300	10						223	[48]	
	extruded	350	1						130	[48]	
	extruded	350	5						163	[48]	
	extruded	350	10						176	[48]	
	extruded	400	1						87	[48]	
	extruded	400	5						116	[48]	
	extruded	400	10						126	[48]	
	T5	130	1d-3	258.4	186.1			42.8		[91]	forged at 330 °C with 1 mm$s^{-1}$, artificially aged at 150 °C for 30 h, sample in web position
	T5	130	1d-3	226.8	124.5			30.3		[91]	forged at 330 °C with 1 mm$s^{-1}$, artificially aged at 150 °C for 30 h, sample in flange
HM21A	T5	200		110	90			49 (in 25mm)		[27]	
	T5	315		90	76			37 (in 25mm)		[27]	
	T5	370		76	55			43 (in 25mm)		[27]	
M1A		93		165	121			25		[27]	
		120		145	107			26		[27]	
		150		131	93			31		[27]	
		200		114	69			34		[27]	
		260		83	45			67		[27]	
		315		41	28			140		[27]	
